# Tor1 and CK2 kinases control a switch between alternative ribosome biogenesis pathways in a growth-dependent manner

**DOI:** 10.1371/journal.pbio.2000245

**Published:** 2017-03-10

**Authors:** Isabelle C. Kos-Braun, Ilona Jung, Martin Koš

**Affiliations:** Biochemistry Center, University of Heidelberg, Heidelberg, Germany; National Cancer Institute, United States of America

## Abstract

Ribosome biogenesis is a major energy-consuming process in the cell that has to be rapidly down-regulated in response to stress or nutrient depletion. The target of rapamycin 1 (Tor1) pathway regulates synthesis of ribosomal RNA (rRNA) at the level of transcription initiation. It remains unclear whether ribosome biogenesis is also controlled directly at the posttranscriptional level. We show that Tor1 and casein kinase 2 (CK2) kinases regulate a rapid switch between a productive and a non-productive pre-rRNA processing pathways in yeast. Under stress, the pre-rRNA continues to be synthesized; however, it is processed differently, and no new ribosomes are produced. Strikingly, the control of the switch does not require the Sch9 kinase, indicating that an unrecognized Tor Complex 1 (TORC1) signaling branch involving CK2 kinase directly regulates ribosome biogenesis at the posttranscriptional level.

## Author summary

The yeast *Saccharomyces cerevisiae* must, as a single-celled microorganism, rapidly respond to changes in its environment and carefully balance its energy needs with the available resources. One of the major energy-consuming processes in the cell is the production of ribosomes. Ribosome biogenesis is highly dynamic and complex and requires the coordinated action of myriads of factors and RNAs. It begins with the synthesis of the precursor ribosomal RNA (rRNA), which is concurrently cleaved, modified, folded, and assembled together with ribosomal proteins into mature ribosomal subunits. It is known that in response to depletion of nutrients, yeast quickly represses the transcription of genes encoding rRNAs and ribosomal proteins. In this study, we reveal that yeast also rapidly switches to an alternative rRNA processing pathway, in which the precursor rRNA is cleaved differently and the ribosome biogenesis is arrested at a distinct stage. We demonstrate that the choice between the two alternative pathways is controlled by the target of rapamycin complex 1 (TORC1) and casein kinase 2 (CK2) kinase, but does not require Tap42p and Sch9p, which are currently thought to be the major effectors of TORC1. Our results indicate the existence of a so far unidentified branch of TORC1 signaling regulating ribosomes biogenesis at the posttranscriptional level.

## Introduction

All cells must adapt to a constantly changing environment in order to maintain their intracellular equilibrium and to balance growth with survival. Several signaling pathways monitor concentrations of available nutrients or presence of harmful conditions and switch on or off specific transcriptional programs to ensure the best usage of resources. When essential nutrients become unavailable, cell division is arrested at the G_1_ phase of the cell cycle, cells change metabolism, and prepare for entry into the reversible quiescent/G_0_ state, in which they can survive for decades (reviewed in [1]). Most cells from multicellular organisms are also considered to be quiescent, in the G_0_ phase of the cell cycle, as the end stage of their terminal differentiation.

In yeast, like in other microorganisms, several distinct stages of culture growth can be observed in a rich medium, such as yeast extract-peptone-dextrose (YPD): exponential phase, diauxic shift, postdiauxic phase, and stationary/quiescent phase. During exponential phase, with abundant fermentable carbon source (e.g., glucose), yeast cells use fermentation and divide rapidly. The diauxic shift occurs when glucose becomes depleted from the media and yeast switches to respiratory metabolism concomitantly with a sharp decrease in growth rate. In the subsequent postdiauxic phase, yeast grows at a much slower rate, using respiration to provide energy, and starts to acquire characteristic features of stationary cells until the growth ceases completely and cells become quiescent [2]. Yeast starved of other essential nutrients, such as nitrogen, phosphate, or sulphur, can also enter a quiescent state [3]. An interconnected signaling network of key kinases, the target of rapamycin complex 1 (TORC1), protein kinase A (PKA), sucrose non-fermenting 1 (Snf1p), and phosphate metabolism 85 (Pho85p), monitors the nutrient availability in yeast. While the initial transcriptional response to limitation of individual nutrients differs [4,5], there is a common core program for preparation of entry into quiescence, regardless of the limiting nutrient [1].

TORC1 is a highly conserved multimeric protein kinase that controls cell growth and aging from yeast to human [6]. TORC1 takes its name from the fact that it is rapidly and specifically inhibited by the macrolide rapamycin [7]. Inhibition of TORC1 by rapamycin leads to G1 cell cycle arrest, decrease in protein synthesis and ribosome biogenesis, initiation of a specific transcription program, induction of autophagy, and eventual entry to quiescence (reviewed in [8]). The activity of TORC1 correlates primarily with the availability of carbon and nitrogen sources; however, TORC1 activity is also sensitive to other nutrients and exposure to heat or oxidative and osmotic stress [9]. Its activity is also down-regulated during the diauxic shift during growth in a rich media [10]. In yeast, TORC1 signals primarily through two key branches: (1) by phosphorylation of Sch9p (a yeast ortholog of mammalian S6 kinase) and (2) by phosphorylation of Tap42p or Tip41p that are thought to regulate activity of the PP2A phosphatase [9,11,12]. The Sch9 kinase was described as a master regulator of protein synthesis that controls ribosome biogenesis, translation initiation, and entry to quiescence [9,13]. Sch9p affects activity of all three classes of RNA polymerases required for ribosome biogenesis. It controls recruitment of the core initiation factor Rrn3p to the RNA polymerase I (RNA pol I) machinery and thus synthesis of ribosomal RNA (rRNA) [14,15]. Sch9p also regulates RNA polymerase II (RNA pol II) transcription of ribosomal proteins (RPs) and ribosome biogenesis RiBi genes by controlling activity of several transcription factors and repressors [9,13,16,17]. Active Sch9p promotes activity of the RNA polymerase III (RNA pol III), required for transcription of 5S rRNA and tRNAs, by inhibition of its transcription repressor Maf1p [13,18,19].

**Fig 1:**
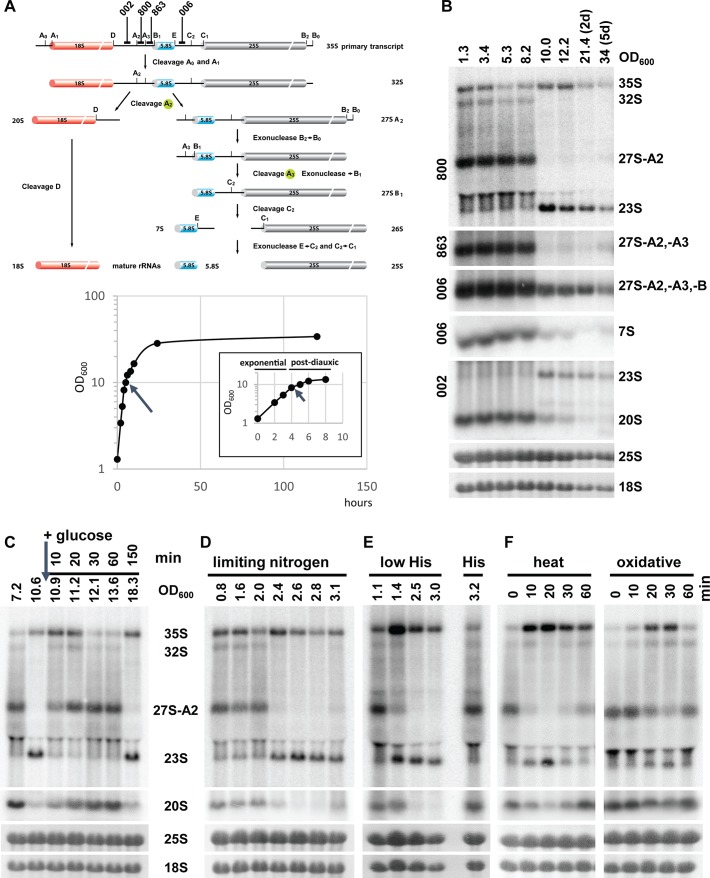
Pre-rRNA processing switches between the A2 and A3 pathways. (A) A scheme of pre-rRNA processing in yeast, simplified from [22]. Positions of oligonucleotides used as hybridization probes are indicated above. (B) Northern blot analysis of the pre-rRNA processing during different stages of growth. Wild-type yeast YMK118 was grown in YPD for up to 5 d. A growth curve with OD_600_ values plotted against time is shown. An evaporation of culture media during extended cultivations likely affected the OD_600_ reading. From our measurement, the error was <20% on day 5. The arrows indicate the time of the switch in pre-rRNA processing. Total RNA was isolated from the same number of cells and analyzed by northern blotting. The probes used for hybridization are indicated left of the gels. The mature 25S and 18S rRNAs were stained with Methylene blue after blotting to a nylon membrane. (C) YMK118 was grown in YPD (2% glucose). After yeast entered the postdiauxic phase, 1% glucose was added (arrow). Samples were harvested at indicated times. Total RNA was analyzed by northern blotting, using the A2–A3 probe. (D) Nitrogen limitation: YMK118 was grown in synthetic dextrose complete (SDC) media with limiting amounts of ammonium sulphate (625 mg/L, i.e., 8-fold less than in standard SDC media). Total RNA was analyzed as in (C). (E) Amino acid starvation; wild-type strain YMK118 was grown in SDC with limiting amounts of histidine (“low His”) (2 mg/L, i.e., 10-fold less than standard SD media). (F) Environmental stress: exponentially growing YMK118 was shifted from 25°C to 37°C or exposed to 0.2 mM Diamide. Total RNA was harvested at indicated times and analyzed as in (C).

Ribosome biogenesis is a major energy-consuming process in the cell, and it is down-regulated in response to nutrient limitation and stress [20]. The complex pathway of ribosome synthesis is best understood in yeast. It starts by the transcription of the 35S pre-rRNA, a large precursor that is cleaved at multiple sites and processed into mature 18S, 25S, and 5.8S rRNAs (Fig 1A), reviewed in [21,22]. The processing of 35S pre-rRNA starts with cleavages at sites A0 and A1, which release the 5′ETS, and is followed by a cleavage at the site A2 that separates the precursors of 40S and 60S ribosomal subunits, 20S and 27SA2 pre-rRNAs, respectively, which are further processed into mature rRNAs. A direct processing at site A3, producing 23S pre-rRNA, has also been observed [23]. Low levels of 23S pre-rRNA are present in wild-type cells but markedly accumulate in ribosome biogenesis mutants, in which they don’t seem to be further processed and are degraded by exosome [22,24]. However, the fate of 23S pre-rRNA remains controversial, and it is usually regarded as a product of aberrant processing [21,22,25]. Interestingly, in higher eukaryotes, the order of early cleavages is often variable, suggesting that multiple processing pathways exist [25].

Here, we show that TORC1 and casein kinase 2 (CK2) kinases control ribosome biogenesis at the posttranscriptional level of pre-rRNA processing. Upon nutrient limitation or exposure to harmful stress, pre-rRNA processing rapidly switches from the standard A2 site cleavage pathway to an alternative pathway using exclusively the A3 cleavage site and thus producing 23S pre-rRNA. Stable isotope labeling with amino acids in cell culture (SILAC) mass spectrometry and in vivo pulse-chase analysis showed that pre-rRNA synthesis continues after nutrients depletion, but the pre-rRNA is processed only into 23S and 27S-A3 pre-rRNAs, and no new ribosomes are made. Inhibition of TORC1 or CK2 kinases induces an identical change in pre-rRNA processing, indicating that TORC1 and CK2 control the choice between the A2 and A3 sites. Analyses of protein composition and phosphorylation changes in preribosomes identified several ribosome biogenesis factors as targets of CK2 kinase and TORC1 pathway. These proteins likely represent the control nodes in ribosome biogenesis.

## Results

### Switch from A2 to A3 pathway during diauxic shift or upon depletion of other nutrients

In exponentially growing yeast, the pre-rRNA processing is initiated by cleavages at sites A0, A1, and A2. We noticed that in yeast cultures undergoing the diauxic shift, rRNA processing at sites A0, A1, and A2 was abruptly abolished, and the pre-rRNA was instead processed at site A3 (Fig 1A and 1B and S1A Fig). The switch in processing was completed within 20 min (S1B Fig). Importantly, the steady-state level of 35S rRNA clearly increased, indicating that its processing was delayed, and the ribosomal DNA (rDNA) transcription was not inhibited. These changes resulted in production and accumulation of the 23S and 27S-B pre-rRNAs; however, the downstream intermediates 20S and 7S pre-rRNA were hardly detectable, suggesting that further ribosome biogenesis was arrested.

The 27SA2 and 23S pre-rRNA are produced from the same primary transcript by a cleavage at sites A2 or A3, respectively, which are separated by only 76 nucleotides. As both 27SA2 and 23S pre-rRNAs are detected by the same hybridization probe, we can directly compare their steady-state levels. Interestingly, the amounts of 27SA2 before shift and 23S pre-rRNAs after shift were comparable, indicating that at least at the initial time points, the change in the processing was reciprocal and indeed represented a “switch” to an alternative processing. The rapid reduction in the 27SA2 levels likely corresponds to its further processing into mature 25S rRNA during the switch in pre-rRNA processing. The 10–20 min required to switch from the A2 to A3 pathway is a long time compared to a short lifetime of the 27SA2 pre-rRNA, which is 15 s or 90 s for cotranscriptionally or posttranscriptionally produced 27SA2, respectively [26]. Similarly, the apparent lack of signal for 27SA3 is due to a short lifetime of this intermediate and its immediate processing to 27SB.

The addition of fresh glucose to a postdiauxic shift culture led to a fast reversal to the pre-rRNA processing using A2 cleavage site within approximately 10 min (Fig 1C). The yeast continued to grow exponentially for about an hour and, upon depletion of the added glucose, switched again to the A3 processing. This behavior was also consistent in synthetic media.

To determine whether the observed switch in pre-rRNA processing is limited only to a carbon source or represents a more general response to a lack of nutrient, we cultured cells under limited nitrogen or amino acid conditions (Fig 1D and 1E). In all cases, the cells reacted in exactly the same manner and changed their pre-rRNA processing abruptly upon depletion of all nutrients tested. We observed identical behavior in three commonly used and well-characterized wild-type strains W303, BY4741, and CEN-PK2 (Fig 1B, S1C Fig). We conclude that yeast cells switch to an alternative ribosome biogenesis pathway in response to limited nutrient availability. For simplicity, in the following text, we will refer to the two alternative processing routes as A2 pathway (occurring in rapidly growing yeast) and A3 pathway (slow or arrested growth).

### Heat shock and oxidative stress activate the switch to A3 pathway of pre-rRNA processing

Ribosome biogenesis is also down-regulated during various cellular stresses. We therefore tested if the switch from A2 to A3 pathway represents a more general mechanism used during the stress response. A wild-type yeast was exposed to a mild heat shock (shift from 25°C to 37°C), osmotic stress (exposure to 1 M Sorbitol), or oxidative stress (0.2 mM Diamide), conditions that were shown to induce a rapid down-regulation of the expression of genes encoding RPs and biogenesis factors [5,27]. As can be seen in Fig 1F, the mild heat shock led to an immediate temporal switch to A3 pathway, with a strong accumulation of 35S pre-rRNA. The processing then returned to normal A2 pathway after about 30 min, after adjustment of the cellular metabolism to the growth at higher temperature. An oxidative stress, caused by exposure of the cell to 0.2 mM diamide, showed a slightly milder temporal switch to A3 pathway (Fig 1F). We used very mild conditions for both stresses, milder than used standardly, in order not to kill or stop cells growing and thus allow the evaluation of their effect on ribosome biogenesis. Harsher oxidative stress might elicit stronger phenotype. The osmotic shock elicited a less clear change to A3 pathway, with noticeable reduction of 27SA2 levels but no obvious increase of 23S pre-rRNA levels (S1D Fig). These results indicate that the switch from A2 to A3 pathway of pre-rRNA processing is a general response to certain environmental stresses when ribosome biogenesis needs to be repressed.

### A3 pathway is nonproductive

**Fig 2:**
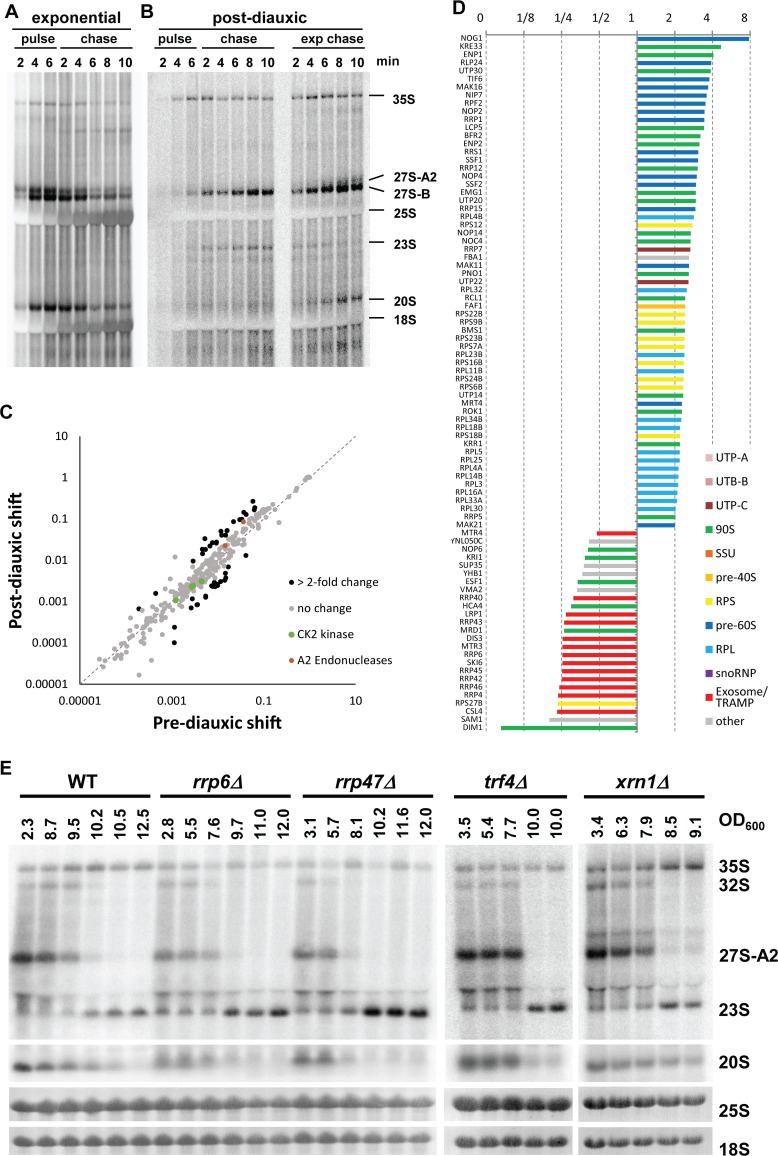
In vivo pulse-chase and proteomic analyses of the pre-rRNA processing in exponential and postdiauxic shift cultures. (A) Low density (OD_600_ = 2) YMK118 was pulse labeled in vivo with ^3^H-uracil for 6 min and then chased with nonradioactive uracil for 10 min. (B) Yeast culture in early postdiauxic phase (OD_600_ = 10) was pulse labeled for 6 min. The culture was split, and one-half was chased for 10 min, as in (A) (on the left). To the other half of the culture, fresh media and no-radioactive uracil was added to the same final concentration and chased for 10 min (right side, “exponential chase”). (C) Comparison of intensity based absolute quantification (iBAQ) values normalized to bait of 324 proteins detected in the affinity purified preribosomes from pre- and postdiauxic cultures. Proteins changed more than 2-fold are in black. (D) Histogram of SILAC Heavy/Light (H/L) ratios (normalized to bait) for proteins with more than 2-fold change in the preribosomes purified from postdiauxic shift versus prediauxic shift cultures (for high resolution see S7 Fig). An experiment with a higher coverage is shown. Underlying numerical data are in S1 Data. (E) Northern blot analysis of RNA isolated form wild-type yeast or strains deleted for different exosome/TRAMP complex factors or Xrn1. All strains were grown until their growth stopped after the diauxic shift. The *xrn1Δ* strain stopped growing at the indicated lower OD_600_ than the other strains.

In the previous experiments, we observed that the 35S pre-rRNA continued to accumulate in cells after nutrient depletion, even when the cells had practically stopped proliferating. In addition, the level of 23S pre-rRNA was comparable to levels of 27SA2 in exponentially growing cells. Furthermore, no 20S pre-rRNA was detectable after the switch, raising the question of whether the 23S rRNA intermediate is further processed and new ribosomes are produced. There are two possible explanations: (a) the pre-rRNA synthesis (transcription and processing) is arrested and preribosomes with either 35S or partially processed pre-rRNA are stalled and waiting for a change in growth conditions; or (b) the rDNA transcription is ongoing, the 35S pre-rRNA continues to be synthesized but is processed at the site A3, and subsequent processing is abolished, and thus no new ribosomes are made. To distinguish between these possibilities, we performed in vivo ^3^H-uracil pulse-chase. The ^3^H-uracil can be incorporated into pre-rRNA only during ongoing transcription. Therefore, any radioactive labeling of the 35S and 23S pre-rRNAs clearly indicates ongoing de novo synthesis. If rDNA transcription is repressed (a), no pre-rRNA should be labeled and detected; whereas, in the case (b), both 35S and other pre-rRNA precursors should become labeled and detectable. Exponential (OD_600_ = 2) or postdiauxic (OD_600_ = 10) cultures were pulse-labeled for 6 min with ^3^H-uracil and then chased with cold uracil for 10 min. As expected, in the exponentially growing cells, all the main pre-rRNA processing intermediates and mature 18S and 25S rRNAs were clearly detectable within the first minutes of labeling (Fig 2A). The pre-rRNAs could be clearly chased by the addition of nonradioactive uracil. In contrast, in the postdiauxic culture, only three RNAs were clearly detectable: 35S, 27S-B, and 23S pre-RNAs. No mature 18S or 25S rRNAs or 27S-A and 20S intermediates were detected (Fig 2B). Interestingly, while the 35S pre-rRNA could be slowly chased with cold uracil, the 23S and 27S-B pre-rRNAs seemed to accumulate in the postdiauxic cells, indicating that they are not processed further. The slow and less-efficient chase is likely partially due to a reuse of the ^3^H-uracil that remains in the intracellular pool of uracil. The ^3^H-uracil from degraded RNAs (e.g., processed spacers of the pre-rRNA or introns) remains in the cell, as it is not efficiently diluted out by the provided cold uracil, especially in very slowly or non-growing cells, as is the case here. This contributes to the observed accumulation of the longer-lived 23S and 27S pre-rRNA that are not processed further. It is also worth noting that the 35S pre-rRNA was detected prior to the appearance of 27S pre-rRNA, revealing that the A3 cleavage in postdiauxic cells occurred posttranscriptionally.

To analyze the effect of fresh media on the postdiauxic cells, one-half of the pulse-labeled culture was diluted with fresh, prewarmed media supplemented with cold uracil to simultaneously initiate a chase (Fig 2B right). The 20S and 27SA2 pre-rRNAs appeared rapidly, and mature 18S and 25S rRNA were detectable after about 6 min. The 35S pre-rRNA was slightly reduced. The 23S pre-rRNA was not detectable after 8 min.

It is important to note that in the postdiauxic culture, the total amount of transcription (radioactive signal) was strongly reduced, in agreement with previous reports [28]. We estimate from the pulse-chase experiment in Fig 2 that the overall transcription in the postdiauxic cells is about 2% of the transcriptional level in the fast exponentially growing cells. While 2% might seem low, rDNA is very highly transcribed, and it represents a very significant number of transcriptional events, comparable to highly expressed protein-coding genes. It is technically difficult and maybe physiologically irrelevant to distinguish if either the repression of rDNA transcription or change in processing occurs first. It is likely and economically logical that cells would down-regulate both the transcription and processing simultaneously.

In the pulse-chase experiment, the signal intensity of 23S pre-rRNA was lower than the signal corresponding to 27S pre-rRNA species. This is seemingly contrary to the results from northern blotting (Fig 1B), in which the 23S pre-rRNA level is clearly equal to 27SA2 pre-rRNA. However, the strong signal in the pulse-chase experiment corresponds to the 27SB pre-rRNA, an intermediate with a longer lifetime accumulating to much higher levels than the 27SA2 pre-rRNA. Nevertheless, the 23S pre-rRNA might also be subject to degradation. We therefore analyzed levels of the 23S pre-rRNA in exosome, TRAMP complex, and Xrn1 exonuclease mutants (Fig 2E). The 23S pre-rRNA levels were clearly increased in the mutants of exosome and TRAMP complex (*rrp6Δ*, *rrp47Δ*, and *trf4Δ*) but not in the *xrn1Δ* strain lacking major 5′-3′ exonuclease. Therefore, the 23S pre-rRNA seems to be turned over by exosome.

These results indicate that the A3-type pre-rRNA processing pathway following the diauxic shift is nonproductive and does not lead to detectable synthesis of new ribosomes. This is also in agreement with the observed reduction of the mature 18S and 25S rRNAs content per cell at later time points after the diauxic shift in Fig 1B (total RNA from the same number of cells were loaded per lane). We cannot formally exclude that a small number of ribosomes (technically undetectable in our experiments) are being synthesized or that ribosomes with potentially distinct properties are made using the A3 pathway at a certain time point (see discussion).

### Early preribosomes composition changes after the switch in pre-rRNA processing

To further understand changes in the ribosome biogenesis after the switch to alternative rRNA processing, we analyzed the protein composition of the early preribosomes (90S preribosomes) by SILAC mass spectrometry. The ribosome biogenesis factor Pwp2p (Utp1p) was used as a bait. Pwp2p is commonly used as a bait in tandem affinity purifications, as it is a stable component of early preribosomes, in which the first pre-rRNA processing steps including the cleavages at the A2 or A3 sites occur [29–31]. It also remains associated with the 23S pre-rRNA produced by cleavage at the A3 site. To confirm that Pwp2p associates with newly formed preribosomes after the diauxic shift, we analyzed the RNA copurifying with Pwp2p (S2 Fig). The purified Pwp2-complexes from postdiauxic shift cells contained increased amounts of 35S/32S and 23S pre-rRNA, with majority (about two-thirds) of Pwp2p associated with 35S and 32S pre-rRNA. This indicates that Pwp2p is recruited to newly formed preribosomes after the diauxic shift and remains bound following the A3 cleavage.

For the SILAC mass spectrometry, an identical number of cells before the diauxic shift (light media) and 1 hr after diauxic shift (heavy media) were harvested, mixed, and lysed. The 90S preribosomes were purified via Pwp2-FLAG-TEV-ProteinA (performing both steps of the tandem affinity purification). The proteins present in the purified Pwp2-associated preribosomes and the relative ratios between the heavy and light labeled cultures were determined by a mass spectrometry (S1 Data). On average 25%–30% decrease in the purification of the heavy labeled Pwp2p bait (from postdiauxic shift culture) was observed. This is in agreement with a slow-down of growth after the diauxic shift. The data was normalized to the H/L (Heavy/Light) ratio of the Pwp2p bait to correct for the reduced purification (the H/L ratio of Pwp2p was set to 1). Fig 2C shows that the overall composition of 90S preribosomes is unaffected, as the relative abundance of the majority of 324 quantified proteins does not change significantly. However, there are the following notable exceptions (Fig 2D). In agreement with the shift from cotranscriptional to posttranscriptional rRNA processing, a number of pre-60S biogenesis factors (dark blue bars) and RPs of the large subunit (RPLs) (light blue) were enriched in preribosomes from the A3 pathway. Several RPs of the small subunit (RPSs) were also enriched. Notably, only the Rps27p (eS27) was reduced. A depletion of the Rps27p was reported to lead to 23S pre-rRNA accumulation [32]. Thus, the loss of Rps27p could represent the mechanism underlying the switch to A3 pathway. However, further data analysis excluded this possibility (see discussion and S8 Fig for more detail). Also, several early preribosome factors (green bars) remained trapped in the Pwp2p particles compared to prediauxic culture. Importantly, two endonucleases Fcf1 and Rcl1 that were reported to mediate cleavage at the site A2 [33,34] remain associated or even enriched in A3 preribosomes (red dots in Fig 2C). Therefore, the loss of the A2 cleavage is not due to the absence of the responsible endonuclease. On the other hand, subunits of the exosome complex, required for the pre-rRNA processing of downstream intermediates, were strongly reduced in the pre-ribosomes from A3 pathway. This is in agreement with the fact that pre-rRNA processing seems to be arrested, and thus substrates for the exosome, which are produced in A2 pathway, are not being made. The methyltransferase Dim1p, which is recruited at later stages of nucleolar processing ([30], was also strongly reduced, indicating that the ribosome biogenesis might be arrested before its recruitment. Interestingly, several early factors (Nop6p, Kri1p, Esf1p, Hca4p, and Mrd1p), all required for A2 cleavage, were also released from 90S preribosomes. These proteins might represent potential regulatory nodes of the switch between the two alternative pathways.

We conclude that although the early steps of ribosome biogenesis continue after the switch to the A3 pathway, and the overall composition of the preribosomes is largely unaffected, the subsequent maturation of preribosomes is arrested after the A3 cleavage.

### TORC1 pathway controls the switch between A2 and A3 pathways

**Fig 3:**
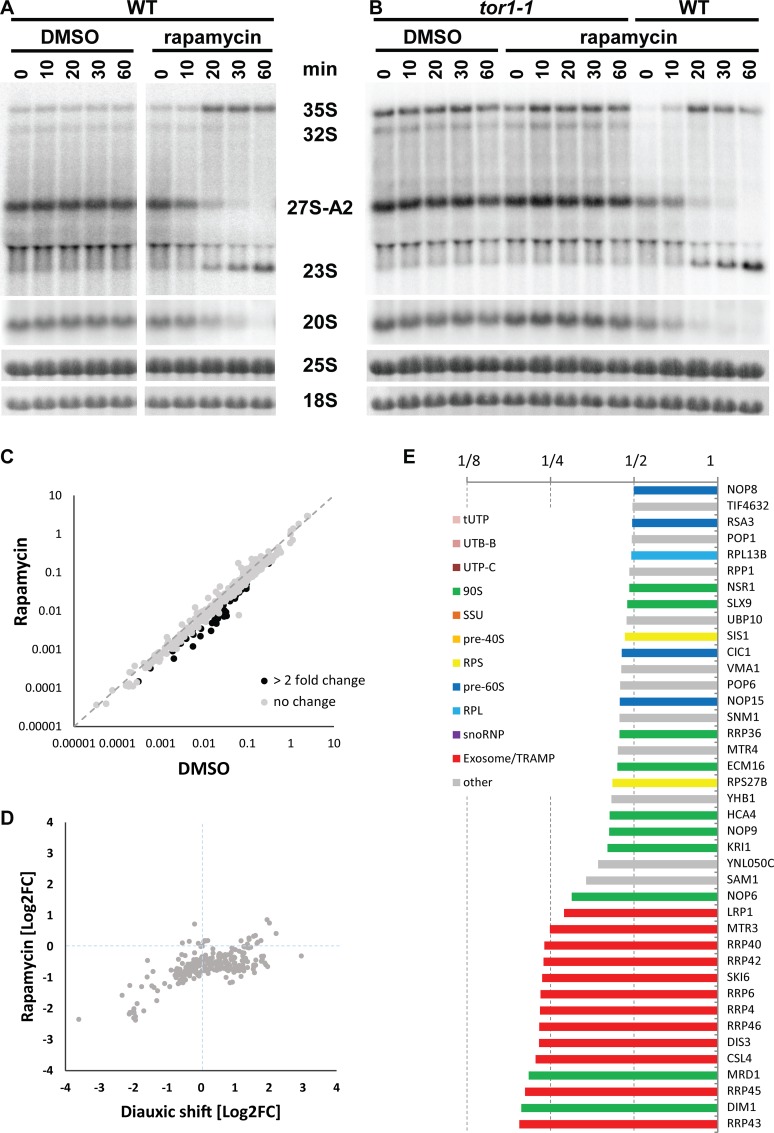
TOR pathway controls the switch between the A2 and A3 pathways. (A) Pre-rRNA processing in YMK118 grown in YPD (OD_600_ = 1.7) and treated with either DMSO or rapamycin, analyzed by northern blotting using probe A2–A3. (B) A rapamycin-insensitive *tor1-1* strain was treated the same as in (A). (C) Comparison of iBAQ values (normalized to bait) of 350 proteins detected in the affinity-purified preribosomes from cultures treated with DMSO or rapamycin. Proteins changed more than 2-fold are in black. (D) Comparison of SILAC H/L ratios (normalized to bait) of proteins from the affinity-purified preribosomes affected either by diauxic shift or by rapamycin treatment. (E) Histogram of SILAC H/L ratios (normalized to bait) for proteins with more than 2-fold change in the preribosomes purified from rapamycin versus DMSO-treated cultures. An experiment with a higher coverage is shown. Underlying numerical data are in S1 Data.

It is well established that TORC1 regulates rRNA transcription by RNA pol I in response to nutrient availability. Therefore, we investigated if the pre-rRNA processing and, in particular, the switch between A2 and A3 pre-rRNA processing pathways is also under the control of TORC1. We inhibited TORC1 in exponentially growing cells by rapamycin and analyzed the pre-rRNA processing. This analysis (Fig 3A) clearly shows that inhibition of TORC1 caused a rapid switch from the A2 to A3 pathway. The production of the 27SA2 pre-rRNA was abolished, whereas the 23S pre-rRNA accumulated instead in rapamycin-treated cells. The levels of 35S pre-rRNA increased, showing that transcription of rDNA continued and that the switch in processing occurs before or concomitantly with down-regulation of rDNA transcription by TORC1. The change in pre-rRNA processing induced by rapamycin was specific to TORC1, as the strain expressing a mutant Tor1-1p, which cannot bind rapamycin, failed to switch to the A3 pathway (Fig 3B).

In yeast, in addition to TORC1, three other core signaling pathways (PKA, PHO85, and SNF1 kinases) are known to contribute to the regulation of cellular metabolism in response to nutrient availability. Interestingly, deletion of either *PHO85* or *SNF1* or inhibition of PKA in an analog-sensitive PKA strain did not affect the cells’ ability to rapidly switch to A3 pathway (S3 and S4 Figs). Taken together, these findings demonstrate that the regulation of A2 to A3 switch in pre-rRNA processing is specific to TORC1 pathway.

It is well established that nutrient depletion or rapamycin treatment also causes the down-regulation of RP genes transcription [28,35]. We analyzed the mRNA levels of several RP genes by northern blotting in various conditions used in this study (S5 Fig). Our data shows that the transcription of RP genes is down-regulated simultaneously with the switch of pre-RNA processing to A3 pathway (see discussion).

The protein composition of preribosomes after inhibition of TORC1 by rapamycin was analyzed by SILAC in an identical way as in Fig 2. Exponentially growing cultures at OD_600_ = 2 were treated for 20 min with rapamycin (heavy media) or DMSO only (light media). The preribosomes were isolated via the affinity-tagged Pwp2p. A 25%–30% reduction of bait purification from the rapamycin-treated culture was observed, in agreement with a growth arrest by rapamycin. As can be seen from Fig 3C, the overall composition of preribosomes is unaffected, analogous to the diauxic shift. However, in contrast to the diauxic shift, rapamycin treatment led to a general reduction of copurified ribosome biogenesis factors (Fig 3D and 3E). This corresponds to distinct effects of the rapamycin treatment versus diauxic shift on cell growth. While postdiauxic cells continue to slowly grow, rapamycin rapidly arrests cell proliferation, leading to the observed reduction of ribosome biogenesis. Nevertheless, there are several important parallels between the two conditions, as the same proteins were clearly reduced in preribosomes after either rapamycin treatment or diauxic-shift. Namely, the exosome complex, Dim1p, and prerequisite factors for A2 cleavage Hca4p, Mrd1p, Kri1p, and Nop6p. This finding strengthens the idea that these factors are implicated in the regulation of the switch between the two pre-rRNA processing pathways. We conclude that the TORC1 pathway controls the choice between the two alternative pre-rRNA processing programs.

### The A2 to A3 switch is not dependent on RNA pol I

**Fig 4:**
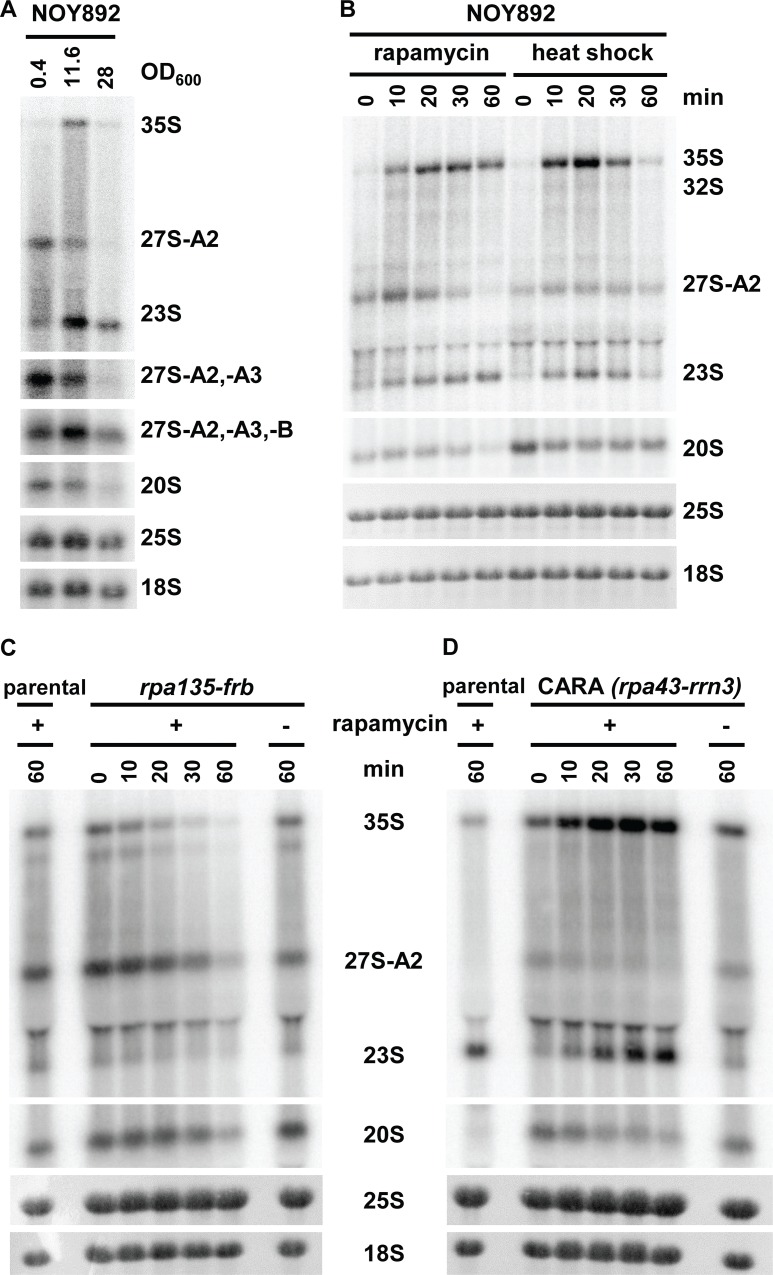
The switch from the A2 to the A3 pathway is not dependent on RNA pol I. (A) Northern blot analysis of the pre-rRNA processing in the strain NOY892, grown in YP-galactose. Hybridization probes as described in Fig 1A and 1B. (B) NOY892 was treated with 200 ng/ml rapamycin at OD_600_ = 2 or exposed to heat shock by shifting from 25°C to 37°C. (C) Northern blot analysis of pre-rRNA processing in the anchor-away strain BEN135, expressing Rpa135–FRB fusion treated with rapamycin at OD_600_ = 2. (D) Northern blot analysis of the pre-rRNA processing in the CARA strain treated with rapamycin OD_600_ = 2.

In the current understanding, TORC1 regulates rRNA synthesis at the transcriptional level by promoting the RNA pol I initiation. Our results indicate that the switch between the two alternative pre-rRNA processing pathways occurs at the posttranscriptional level. We were therefore interested to find out if RNA pol I is required for this regulation. We used the strain NOY892, in which all rDNA repeats are deleted, and the 35S pre-rRNA is transcribed solely from a *GAL7* promoter by RNA pol II [36]. The cells were grown in either rich or synthetic media with galactose as a carbon source. Fig 4A reveals that upon depletion of the carbon source, the pre-rRNA processing switched from A2 to A3 pathway in a fashion identical to wild-type yeast. These results indicate that the change in processing is not dependent on transcription by the RNA pol I.

### The A2 to A3 switch is not a simple consequence of a reduced rate of the pre-rRNA synthesis

The initiation of rDNA transcription by the RNA pol I and thus the synthesis of pre-rRNA is strongly reduced during the diauxic shift or rapamycin treatment. Is it possible that the switch between the A2 and A3 pathways is a simple consequence of changes in the kinetics of pre-rRNA synthesis (such as the number of transcribing RNA polymerases or the elongation rate) and/or imbalance in the levels of pre-rRNA and processing factors? The following experiments were designed to provide an insight into this issue.

The previous experiment with the *GAL7* promoter demonstrated that the switch in the pre-rRNA processing is not specific to the RNA pol I. To further exclude that the observed change in processing is not due to a general down-regulation of transcription from the *GAL7* promoter after depletion of galactose, the exponentially growing strain NOY892 was treated with rapamycin or exposed to a mild heat shock (Fig 4B). In both conditions, 23S pre-rRNA immediately appeared within the first 10 min, concomitant with an increase in 35S pre-rRNA levels. Available transcriptomics data show that the *GAL7* promoter activity is not greatly affected by either nutrient depletion (apart from galactose), heat shock, or rapamycin [5,10]. Thus, the number of RNA polymerases or the elongation rate of RNA pol II does not change significantly in these conditions. These results argue that the switch from A2 to A3 pathway is unlikely to be caused by a reduced number of RNA polymerases passing through the rDNA or a changed elongation rate.

Next, we addressed the question if a cessation of RNA pol I transcription would induce a change in the pre-rRNA processing. We used an anchor-away strain BEN135 [37,38], in which Rpa135, the second largest subunit of RNA pol I, was fused to FKBP−rapamycin-binding (FRB) domain, and large subunit RP Rpl13Ap was fused to FKBP12. The strain is resistant to rapamycin, and the addition of rapamycin induces dimerization of FKBP12 and FRB domains and, thus, sequesters the Rpa135p in the cytoplasm, effectively abolishing transcription by RNA pol I within minutes. Importantly, the reduced availability of Rpa135p affects the number of RNA pols I available for transcription but not the elongation rate of the transcribing polymerases. Fig 4C shows that the level of all pre-rRNA species reduced equally without a change in the cleavage pattern, confirming that the switch from A2 to A3 pathway is independent of the rate of transcription.

Finally, we also asked whether continued robust transcription of pre-rRNA would interfere with the A2 to A3 switch in processing. We took advantage of the CARA (Constitutive Association of Rrn3 and A43) strain, in which RNA pol I subunit Rpa43p is fused with the transcription factor Rrn3p, rendering it resistant to rapamycin [39]. In the CARA strain treated with rapamycin, the RNA pol I is not inhibited, but other cellular processes remain affected by the rapamycin treatment. Therefore, using the CARA strain, we can separate the effects of rapamycin on pre-rRNA processing from transcription. We treated exponentially growing culture of the CARA strain with rapamycin and analyzed the pre-rRNA processing (Fig 4D). The cells accumulated large levels of both 35S and 23S pre-rRNA, corresponding to the usage of the A3 pathway. The ongoing strong pre-rRNA synthesis did not affect the switch in pre-rRNA processing.

Our findings provide a strong evidence that the A2 to A3 switch in pre-rRNA processing is neither dependent on the RNA pol I nor likely caused by a change in the elongation rate or the number of transcribing polymerases.

### Sch9 kinase is dispensable for A2 to A3 switch

**Fig 5:**
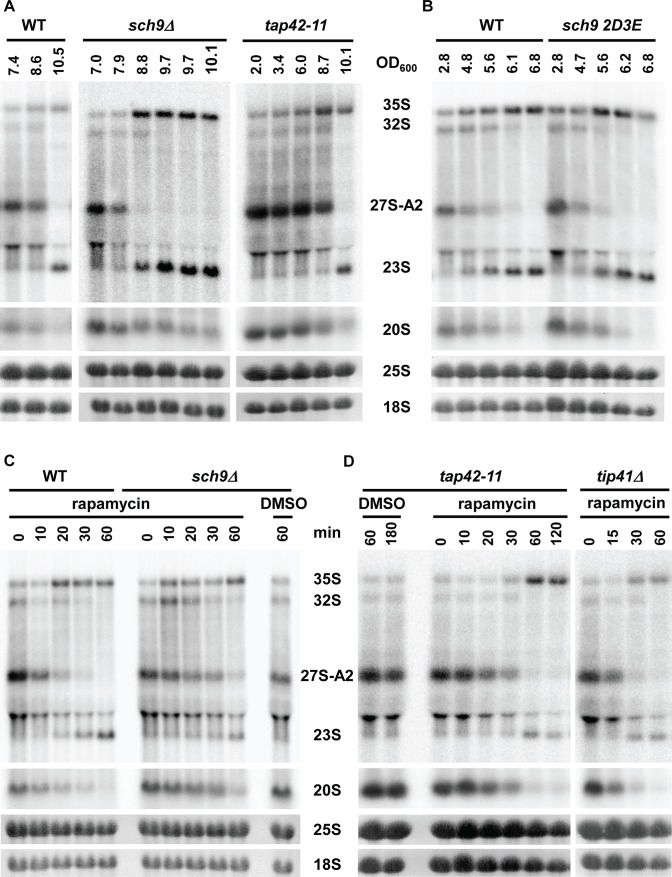
Role of Sch9, tap42, or tip41 in the A2 to A3 switch in pre-rRNA processing. (A) Pre-rRNA processing before and after diauxic shift in the yeast strain lacking Sch9 kinase (*sch9Δ*) or in the *tap42-11* mutant. The OD_600_ values of harvested samples are shown above the lanes. Northern blot using the A2–A3 probe. (B) Pre-rRNA processing before and after diauxic shift in the strain expressing the hyperactive mutant Sch9-2D3E grown in SDC media. Samples were harvested at indicated OD_600_, and total RNA was analyzed by northern blotting using probe A2–A3. (C) The *sch9Δ* or wild-type strains were treated by rapamycin at OD_600_ = 2, and total RNA was isolated at indicated time points and analyzed by northern blotting (probe A2–A3). (D) The *tap42-11* mutant and *tip41Δ* strain were treated with rapamycin as in (C).

Sch9, the yeast ortholog of the mammalian S6 kinase, was described as a master regulator of ribosome biogenesis in yeast, regulating transcription by all three classes of RNA polymerases [9,13]. We tested if Sch9 also controls the switch between A2 and A3 pathways. A yeast strain deleted for Sch9 (*sch9Δ)* was grown until the growth slowed down after the diauxic shift. Fig 5A demonstrates that in the absence of Sch9p, the pre-rRNA processing also changed rapidly from A2 to A3 pathway. Treatment of the exponentially growing *sch9Δ* strain with rapamycin also elicited a switch from A2 to A3 pathway (Fig 5C). Furthermore, expression of a Sch9-2D3E mutant, which mimics phosphorylated Sch9p and thus remains hyperactive [9], neither prevented nor induced a premature switch in processing (Fig 5B). Identical results were also obtained in a P*tetO7-Sch9* strain, in which the wild-type Sch9p protein (expressed under the control of the tetracycline tetO7 promoter) was depleted for 12 hr (S6 Fig), thus excluding the possibility of suppressor mutations in the *sch9ΔΔ*strain, masking its role in the A2 to A3 switch. We also tested a number kinases or factors acting downstream of Sch9p that were implicated in the regulation of ribosome biogenesis (Yak1p, Atg1p, Whi2p, Sfp1p, Sic1p, Dot6p, Tod6p, Mpk1c, Rim15p). Deletion of none of these factors affected the switch in the pre-rRNA processing (S3 Fig). These findings imply that Sch9p function is dispensable for the control of the choice between A2 and A3 pathways.

### A2 to A3 switch is not affected in *tap42-11* or *tip41Δ* strains

The second major branch of TORC1 signaling pathway is represented by Tap42p and its negative regulator Tip41p. We first attempted to assess the role of Tap42p using a temperature-sensitive *tap42-11* mutant. Unfortunately, the heat-shock due to transfer of the *tap42-11* strain from permissive to nonpermissive temperature affects ribosome biogenesis (see Fig 1F); therefore, this experiment cannot be interpreted. Interestingly, the *tap42-11* strain is partially resistant to rapamycin at permissive temperature. Furthermore, a yeast strain lacking Tip41p was also shown to be partially resistant to rapamycin [40–42]. We therefore assessed if the pre-rRNA processing switches to A3 pathway either during diauxic shift or upon rapamycin treatment. Fig 5A (right panel) shows that *tap42-11* grown at permissive temperature to high density, switches to A3 pathway during the diauxic shift in an identical manner as the control wild-type strain. Similarly, the absence of Tip41p does not affect the switch to A3 pathway in high-density culture (S3 Fig). A treatment of exponentially growing low-density culture by rapamycin induced a switch to A3 pathway in both *tap42-11* and *tip41Δ* strains (Fig 5D).

### CK2 activity is required for A2 to A3 switch regulation

**Fig 6:**
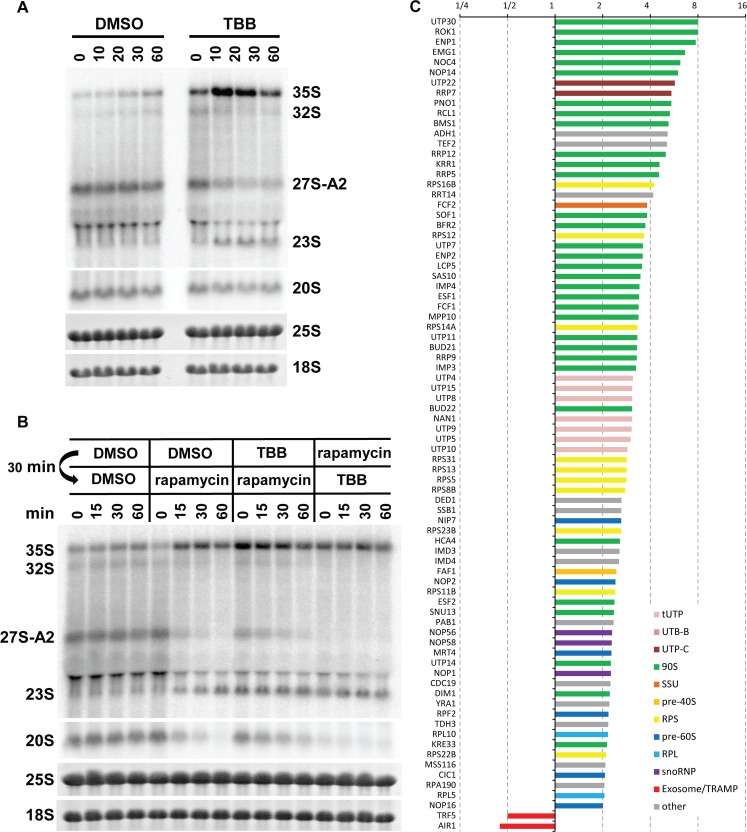
TBB induces a switch between A2 and A3 pathways. (A) Strain YMK118 was treated with the CK2 kinase inhibitor TBB at OD_600_ = 2, and samples were harvested at indicated times. Northern blotting using the probe A2–A3. (B) YMK118 was sequentially treated with inhibitors at OD_600_ = 2 as indicated for 30 min. Then, a second inhibitor was added, and samples were harvested at indicated times. Total RNA was analyzed by northern blotting (probe A2–A3). (C) Histogram of SILAC H/L ratios (normalized to bait) of proteins with >2-fold change in preribosomes from TBB treated cells. The average of two experiments is shown. Underlying numerical data are in S1 Data.

In our preribosome purifications, we detected readily all subunits of CK2 kinase and found that their abundance does not change during diauxic shift (Fig 2C, green circles). The protein kinase CK2 is an essential kinase, the activity of which is required for growth and proliferation. The CK2 kinase is a component of early preribosomes, as a member of the UTP-C subcomplex [43]. It is also a component of a separate CURI complex (CK2, Utp22p, Rrp7p, and Ifh1p), which was implicated in transcriptional regulation of RP genes [35,44]. While the regulation of CK2 activity is largely unclear, it has been recently reported that the CK2 regulatory subunit Ckb1 can be phosphorylated by TORC1 pathway [45]. We therefore assessed whether the CK2 activity is required for the control of the A2 to A3 switch. Wild-type, low-density, exponentially growing cells were treated with 4,5,6,7-Tetrabromobenzotriazole (TBB), a highly selective CK2 inhibitor [46]. The TBB treatment led to an immediate appearance of 23S pre-rRNA and a strong decrease of 27SA2 levels concomitant with the accumulation of 35S pre-rRNA, showing kinetics similar to that of rapamycin treatment (Fig 6A). Interestingly, although the 27SA2 pre-rRNA was strongly reduced, it was not eliminated completely. Sequential treatment of cells by TBB first, followed by rapamycin, showed that the lack of CK2 activity delays the disappearance of 27SA2 pre-rRNA induced by rapamycin treatment (Fig 6B). The activity of CK2 is most likely also required for further 27SA2 processing, and, thus, the inhibition of CK2 delays the disappearance of this species upon rapamycin treatment.

Recently, the nonessential kinase Kns1p was identified as an intermediary between TORC1 and CK2 [45]. We assessed whether Kns1p is required for the A2 to A3 switch. The yeast strain *kns1Δ* lacking Kns1p was grown into high density, and pre-rRNA processing was analyzed by northern blotting (S3 Fig, top right). The switch in pre-rRNA processing from A2 to A3 pathway occurred in an identical fashion to wild-type strains, revealing that the Kns1 kinase is not involved in the regulation of the switch in pre-rRNA processing.

We have also analyzed the effect of inhibition of CK2 activity by TBB on the protein composition of preribosomes by SILAC mass spectrometry. Exponentially grown yeast cultures were treated with DMSO (light) or TBB (heavy) for 15 min, and Pwp2-containing preribosomes were purified. Two independent experiments were performed with two different lots of TBB. Perhaps due to the different lot of TBB used, the second experiment showed similar but smaller changes in the protein composition than the first experiment (S1 Data). In both cases, about 2-fold change in the purification of the bait protein Pwp2p was observed, presumably due to a strong arrest of cell growth by TBB. The H/L ratios of individual proteins were normalized to bait. Fig 6C shows that TBB treatment leads to a robust enrichment of early 90S factors in Pwp2-complexes. Interestingly, Utp22p and Rrp7p, components of the CURI complex, were also strongly enriched. In addition, all four subunits of the CK2 kinase were identified and enriched (although Cka1p, Cka2p, and Ckb1p were quantified only in one of the two replica experiments; S1 Data). Importantly, the 90S factors Hca4p, Mrd1p, Esf1p, Nop6p, and Kri1p, which were strongly reduced in preribosomes from postdiauxic shift or rapamycin-treated cultures, were enriched following CK2 inhibition by TBB. The contrasting behavior of these factors in the three conditions that all cause the switch to A3 pathway makes these factors unlikely candidates to directly regulate the choice between the A2 and A3 cleavage sites. In summary, the CK2 kinase inhibition by TBB produces overlapping but not identical phenotype to diauxic shift or rapamycin treatment.

Added together, these results show that CK2 kinase activity is required for A2 pathway pre-rRNA processing and at least partially for control of the choice between A2 to A3 cleavage sites. It most likely acts downstream of TORC1.

### Protein phosphorylation changes induced by diauxic shift, rapamycin, or TBB

Our results suggested that ribosome biogenesis is directly regulated by TORC1 pathway and CK2 kinase at the posttranscriptional level. Several proteomic studies analyzed rapamycin-sensitive phospho-proteome in yeast [13,47,48]. We mined the data for ribosome biogenesis factors. Surprisingly, only a relatively small number of ribosome biogenesis factors showed significant phosphorylation changes, and these changes in the levels of phosphorylation varies widely between the reports (S1 Table). These studies analyzed whole proteomes and, therefore, might not have detected changes present in different complexes, such as preribosomes. We therefore compared protein phosphorylation pattern of the Pwp2-preribosomes before and after the diauxic shift, rapamycin, or TBB treatment. We identified several hundred phosphorylation sites (S1 Data). Most identified sites were detected in previous phosphoproteome studies (PhosphoGRID, [49], and S1 Table). Interestingly, we observed that larger changes in phoshorylation were induced by the diauxic shift and TBB compared to rapamycin treatment, which elicited less than 2-fold changes, similar to the results from the above-mentioned previous studies (S1 Data and S1 Table). Importantly, the phosphorylated proteins identified are overlapping between the three conditions. However, the identified phosphorylation sites often differ between experiments, showing that our analyses are not saturating, and not all existing phosphorylation sites were identified.

**Fig 7:**
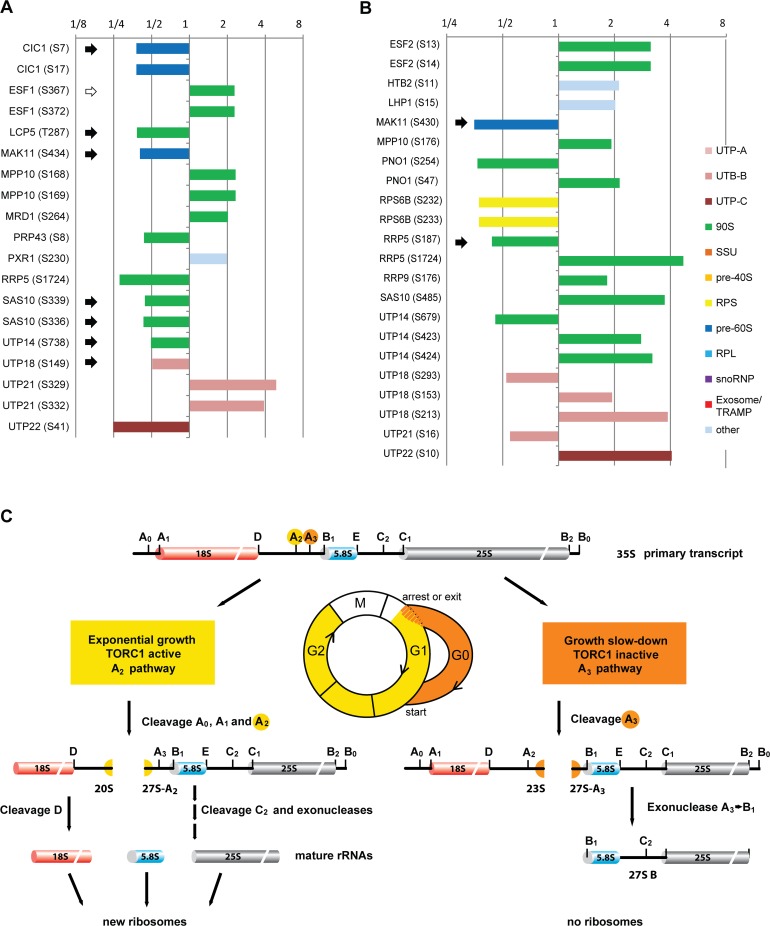
Protein phosphorylation changes during diauxic shift. (A) Phosphorylation sites changed more than 2-fold during diauxic shift. The phosphorylation changes are represented by colored bars. The black arrows indicate sites with the CK2 kinase’s phosphorylation consensus sequence. (B) Phosphorylation sites changed >1.8-fold after TBB treatment. The black arrows indicate sites with the CK2 kinase’s phosphorylation consensus sequence. Underlying numerical data are in S1 Data. (C) Updated model of pre-RNA processing in yeast.

Proteins and their modification sites that changed at least 2-fold during diauxic shift are plotted in Fig 7A. The phosphorylation of some of these proteins (Cic1p, Esf1p, Sas10p, and Utp14p) were also found to be affected by rapamycin in previous studies (S1 Table) but not in our experiments (S1 Data). Significantly, the majority of the affected proteins are known to be prerequisites for the cleavage of pre-rRNA at the site A2. However, as all are components of the early preribosomes, it is not clear if they play mechanistically a direct role in the process. Their phosphorylation might be required for proper and directional preribosome assembly but not for cleavage per se. Nevertheless, these proteins are good candidates for the pre-rRNA processing regulator.

Importantly, we identified a clear loss of phosphorylation of multiple proteins at predicted CK2 kinase consensus sites (x-S/T-xx-E/D/pS/pY) [50–52] (black arrows in the Fig 7A). This is in a good agreement with the observed rapid loss of A2 cleavage and switch to the A3 pathway after inhibition of the CK2 kinase by TBB. To assess if any of these proteins might be direct substrates of CK2, we analyzed Pwp2-containing preribosomes from cells treated with TBB (Fig 7B and S1 Data). A majority of the proteins that showed at least 1.8-fold change in phosphorylation after TBB treatment are among the proteins changed during the diauxic shift, specifically, Mak11p, Mpp10p, Rrp5p, Sas10p, Utp14p, Utp18p, Utp21p, and Utp22p. Intriguingly, phosphorylation of Utp22p, which is a component of the CURI complex containing CK2 kinase itself, was strongly increased at Ser10 (not a CK2 consensus site). It is unclear if CK2 activity controls phosphorylation at this site. In the other factors, we identified increase or decrease of phosphorylation mostly at sites different from the phosphorylation sites identified in the diauxic shift experiment. There are two notable exceptions. One of them is Mak11p, phosphorylation of which was reduced to a similar extent in both the diauxic shift and TBB treatment at sites Ser434/430, which are part of a CK2 consensus site. Mak11p is involved in early steps of 60S biogenesis. Therefore, it might regulate 60S maturation after diauxic shift. The other exception is Rrp5p, which showed reduced phosphorylation at Ser187, which lies directly adjacent to CK2 consensus. In addition, phosphorylation of Rrp5p was also changed at Ser1724, identified in both diauxic shift and TBB experiments. Rrp5p is a huge protein proposed to coordinate pre-rRNA processing and assembly of early preribosomes [53]. It is thus an ideal candidate for regulation of the A2 and A3 cleavage choice.

Our results indicate that a subset of the phosphoproteins identified in the diauxic shift experiment are directly or indirectly under the CK2 control; however, with the exception of Mak11p and perhaps Rrp5p, they do not represent direct bona fide CK2 substrates. Future research will also reveal if Mak11p and Rrp5p represent regulatory nodes of the TORC1 pathway controlling the pre-rRNA processing on a posttranscriptional level.

## Discussion

In this study, we show that TORC1 and CK2 kinases regulate pre-rRNA processing at the posttranscriptional level in response to nutrient availability or stress. Ribosome biogenesis clearly switches between two alternative processing routes, the A2 and A3 pathways. Such changes in the pre-rRNA processing occurs, for example, during the postdiauxic growth phase before entry to quiescence or during a transient arrest of ribosome biogenesis upon exposure to stress such as a heat shock. Based on the results presented here, we propose an updated model of the pre-rRNA processing and ribosome biogenesis in yeast (Fig 7C).

During a rapid exponential growth, TORC1 is active and yeast uses the A2 pathway to produce mature ribosomes. However, under unfavorable conditions, such as depletion of nutrients or exposure to environmental stress, the growth slows down sharply, or cells are transiently arrested in G1. TORC1 becomes inactive, leading to a switch in pre-rRNA processing to the non-productive A3 pathway. The 35S pre-rRNA cleavage pattern changes from the A2 to the A3 cleavage site, concomitant with the abolition of 5′ETS and further downstream processing. This leads to accumulation of the 23S and 27S-B pre-rRNAs without further detectable production of new ribosomes. The A3 pathway continues to be used for several days after depletion of carbon source during the preparation for entry to quiescence (G_0_). When conditions improve, e.g., after addition of fresh glucose to media or adjustment of the cell metabolism for higher growing temperature, the pre-rRNA processing reverts rapidly to A2 pathway.

The presence of low levels of 23S pre-rRNA in wild-type yeast was reported almost 20 y ago [23], but its physiological function has not been understood and has remained controversial in the field, with most considering it to result from aberrant processing [21,25]. Recently, during preparation of this manuscript, an independent study also observed the appearance of 23S pre-rRNA in high-density cultures or following a treatment with rapamycin [54]. The detection of 23S in exponentially growing yeast cultures seems to be dependent on their handling. Yeast cultures typically are not synchronized and thus contain cells in different stages of cell cycle or age. It is likely that the observed low amount of 23S pre-rRNA is produced in a small percentage of cells that are not growing fully exponentially, perhaps after dilution of a stationary culture or in cultures close to diauxic shift. Our results demonstrate that the 23S pre-rRNA is a physiological intermediate produced when ribosome production is repressed.

There is an important difference between the two situations in which 23S pre-rRNA is observed. The appearance of 23S pre-rRNA was most often reported as a consequence of a depletion of early ribosome biogenesis factors or certain early RPSs [32]. However, the depletions were performed in exponentially growing cells in which all other factors remain available, a condition clearly different from the physiological diauxic shift analyzed here. In cases in which the 23S pre-rRNA was observed as a consequence of a mutation or depletion, only the 18S rRNA processing pathway is usually affected, and 25S rRNA continues to be synthesized, leading to an imbalance of the subunits levels. In contrast, we show here that the production of 23S pre-rRNA after diauxic shift or other nutrient depletion is associated with simultaneous arrest of both 18S and 25S rRNA. This strongly suggests an existence of a mechanism that coordinates the synthesis of both subunits under these undesirable but physiological conditions. We would also like to point out that depletions often were (and still are) performed for 12 hr or more, by which time the cells had very likely undergone the postdiauxic shift and started to accumulate 23S pre-rRNA. Therefore, results of long depletions need to be evaluated with caution.

The switch from A2 to A3 pathway was rapid and completed within 20 min (Fig 1 and S1 Fig). It is worth noting that our yeast cultures are asynchronous, and, therefore, the observed changes represent an average behavior from a large number of individual cells. The actual switch in the processing is likely to be significantly faster in the individual cells. This is corroborated by a more rapid reversal from A3 to A2 pathway upon addition of glucose (10 min). Cells starved for glucose are presumably more synchronized and, therefore, react more uniformly to the addition of fresh nutrient.

The fast kinetics of the switch in processing strongly suggest a direct regulation of the A2/A3 cleavages, e.g., by the phosphorylation or another posttranslational modification of a responsible biogenesis factor. However, two possible alternative mechanisms are discussed below.

The initiation of transcription by the RNA pol I is down-regulated when nutrients are depleted or upon a treatment with rapamycin. We estimate from results of the pulse-chase experiments that rDNA transcription is reduced approximately 50-fold. This means that either only a small number of RNA polymerases initiate pre-rRNA synthesis or that the elongation rate of transcription is strongly reduced. The maturation of pre-rRNA and its assembly into ribosomes is immensely complex and dynamic. It is conceivable that the observed switch in pre-rRNA processing could be an unspecific “side effect” of the down-regulation of RNA pol I transcription. Changes in the initiation and/or elongation rates could, together with an imbalance of ribosome biogenesis factors, affect the folding and assembly and thus the cleavage pattern of the pre-rRNA. The experiments presented in Fig 4 ABCD provide evidence that this is unlikely to be the case. Firstly, the switch from the A2 to A3 pathway was not dependent on the RNA pol I (Fig 4A and 4B). The processing of the pre-rRNA transcribed by the RNA pol II from the *GAL7* promoter switched to the A3 pathway after heat shock or rapamycin treatment, conditions that do not significantly affect the transcription from the *GAL7* promoter. The RNA pol II is not generally down-regulated during nutrient depletion. The *GAL7* promoter also remains active during heat shock and rapamycin treatment [5,10]. Therefore, the switch also occurs in conditions in which the initiation and elongation rates are not significantly changed. Secondly, a reduction of the number of available RNA pol I (but not its elongation rate) by a rapid sequestering of the core subunit Rpa135p in the cytoplasm led to a sharp overall drop in pre-rRNA levels but did not induce a change in the processing pattern (Fig 4C). Thus, a reduction of pre-rRNA levels leading to an imbalance between pre-rRNA and ribosome biogenesis factors did not affect the processing. Thirdly, in the reverse case, the continued synthesis of pre-rRNA in the CARA strain treated with rapamycin (Fig 4D) did not prevent switching to the A3 pathway. Although we cannot formally exclude that a specific change in the elongation rate (or pausing) of the RNA pol I in response to nutrient depletion is responsible for the switch to A3 pathway, such an effect would clearly also represent a case of a specific regulation. Taken together, our observations suggest that the switch between the A2 and A3 pathways is a specific regulatory event, most likely controlled at the posttranscriptional level.

We also need to address another alternative mechanism: the change in pre-rRNA processing being caused by a general unavailability of RPs following nutrient depletion or rapamycin treatment. It is well known that transcription of genes encoding RPs is down-regulated in response to nutrient depletion [28,35]. It has been observed that depletion of several early RPSs leads to pre-rRNA processing defects and accumulation of 23S pre-rRNA [32]. Could reduction of the overall levels of RPs or a specific RP be responsible for the switch to A3 pathway? Ju and Warner reported that the repression of rDNA transcription appeared to precede the reduction of transcription of RP genes [28]. It seems to suggest that cells ensure that the relative levels of RPs and synthesized pre-rRNAs remain balanced, presumably to avoid ribosome assembly defects observed in the depletion experiments mentioned above. Interestingly, in the case of TBB treatment, the levels of RPs’ mRNAs seem to be only partially reduced 15 min after the TBB exposure, while the change in pre-rRNA processing had already completed (S5 Fig and Fig 6A). Crucially, the SILAC analyses of changes in the composition of preribosomes after the diauxic shift and rapamycin and TBB treatments convincingly exclude this alternative mechanism (Figs 2D, 3E and 6C and S8 Fig and S1 Data). The data clearly show that there is no relevant loss of RPSs in the preribosomes after the switch to A3 pathway. On the contrary, most of the copurified RPSs are, in fact, enriched in the preribosomes. The only exception was the Rps27p, which was lost from postdiauxic preribosomes. However, the same protein was enriched in preribosomes after TBB treatment, which also underwent the switch to A3 pathway. Therefore, the Rps27p is not the cause of the switch, and its loss in the diauxic shift is a consequence of other structural changes in the preribosomes. Critically, we identified all the RPSs, depletion of which causes the appearance of 23S and a block in 20S production [32] (S8 Fig). Depletion of 60S processing factors generally does not cause 23S phenotype. Furthermore, no 23S phenotype upon depletion of RPLs was reported in the extensive study by [55]. For completeness, we show also the general but insignificant (less than 2-fold) reduction of RPSs following the rapamycin treatment, in agreement with growth arrest (S8 Fig). However, rapamycin does not fully reflect the physiological conditions of diauxic shift or nutrient depletion. Nevertheless, even in this case, none of the RPSs could be singled out as being more strongly reduced than others. Taking all this data together, the hypothesis that the observed switch in pre-rRNA processing is due to unavailability of RPs can be rejected.

A regulation of late nucleoplasmic steps of ribosome maturation by the Tor pathway has been previously reported for 60S subunit synthesis [56]. The authors found that rapamycin treatment of yeast cells causes sequestration of the Nog1p ribosome biogenesis factor in the nucleolus and cessation of 60S ribosomal subunits maturation. Interestingly, our proteomic analyses show that Nog1p is strongly (~8-fold) enriched in the Pwp2p containing preribosomes, following the postdiauxic shift. This observation agrees nicely with the reported nucleolar sequestration of Nog1p. We did not detect a phosphorylation of Nog1p in our proteomic analyses. It remains unclear whether Nog1p plays a more direct role in the Tor pathway-controlled arrest of ribosome biogenesis at the early stages during diauxic shift.

Neither deletion of Sch9 nor expression of the hyperactive Sch9-3D2E mutant affected cells ability to switch from the A2 to A3 pathway. This is an important observation, as Sch9 is currently considered to be the master regulator of ribosome biogenesis downstream of TORC1, controlling transcription by all three RNA polymerases [9]. It indicates that another, Sch9-independent branch or a direct target of TORC1 is responsible for the regulation of the A2 to A3 switch. A similar case of a partial regulation by Sch9 has been reported [19]. While Sch9 is sufficient to control transcription initiation of RNA Pol III by the phosphorylation of Maf1 repression factor, it has been shown that TORC1 can also directly phosphorylate Maf1 and, thus, partially sidestep the requirement for Sch9 [19]. In the presented work we tested all kinases and factors implicated in ribosome biogenesis acting downstream of Sch9 for their role in the A2 to A3 switch (S3 Fig). The fact that they were all dispensable further supports the existence of a Sch9-independent branch of TORC1 required for the regulation of pre-rRNA processing and ribosome biogenesis. Our data also reveals that the A2 to A3 switch functions normally in *tap42-11* and *tip41Δ* strains. Therefore, Tap42p/Tip41p functions disrupted by the mutations in tap42-11p or by a deletion of Tip41p are not involved in the control of A2/A3 cleavages. This is intriguing, as both major branches of TORC1, Sch9p and Tap42p, do not seem to be involved in the switch regulation; however, we cannot exclude a possible redundancy.

We found that CK2 activity is a prerequisite for the A2 pathway. Inhibition of CK2 led to an immediate switch to the A3 pathway. Prior inhibition of CK2 kinase delayed the effect of rapamycin on pre-rRNA processing, indicating that CK2 most likely lies downstream of TORC1. The role of CK2 in the regulation of pre-rRNA processing is corroborated by our observation of reduced phosphorylation of Serine sites within a CK2 consensus motif in Mak11p and Rrp5p (Fig 7A and 7B). The CK2 kinase is not only a component of preribosome but also forms independent CURI complex with Utp22p, Rrp7p, and Ifh1p that is proposed to be involved in transcription regulation of RP genes and could potentially coordinate it with ribosome biogenesis [35,44]. Interestingly, we did not observe loss of Utp22p or Rrp7p from preribosomes; in fact, both factors were enriched in preribosomes from postdiauxic shift and TBB-treated cells (Figs 2D and 6C and S1 Data). Furthermore, Utp22p was hyperphosphorylated at Ser10, which is not a consensus site for CK2 kinase. Does CK2 activity control phosphorylation of Utp22p at this site by other kinase(s)? Future studies will reveal whether the role of CK2 in CURI complex is independent from its regulation of pre-rRNA processing presented here.

The intermediates 23S or 27SB pre-rRNAs generated after the switch to the A3 pathway appear not to be further processed to mature rRNAs. This is strongly supported by the reduced levels of mature rRNAs in cells after the diauxic shift (Fig 1B, bottom; note that total RNA from the same number of cells was loaded per lane). After the diauxic shift and change to A3 pathway, which occurred at OD_600_ ~ 9.5–10 in YPD, the cells grew very slowly and doubled after 2 d. At this time point, the 25S and 18S rRNA levels were reduced to about half, perfectly fitting with the idea that while the cells continue to grow, they do not produce new ribosomes and, thus, the existing ribosomes are divided between the mother and daughter cells. Whether the 23S and 27SB pre-rRNA produced by the A3 pathway can reenter the productive line after improvement of conditions (fresh nutrients) remains unclear. On the one hand, we saw a reciprocal disappearance of 23S and appearance of 20S pre-rRNAs in the pulse-chase experiment that could indicated that 23S is being processed into 20S pre-RNA (Fig 2A). On the other hand, the 20S could also be directly produced from prelabeled 35S pre-rRNA that is also accumulating while the 23S is subjected to turnover. Unfortunately, the fast kinetics of the pre-rRNA processing prevented us to answer this intriguing question conclusively.

Why do cells continue to synthesize and partially process pre-rRNA if they do not seem to make new ribosomes? Yeast is a unicellular microorganism, and, as such, it must react fast to environmental changes, in both directions—negative (e.g., lack of nutrients when rain washes it away) or positive (e.g., it falls on a new ripe fruit). In nature, it needs to compete with other microorganism for the available resources. Therefore, the faster it can start using resources and grow, the more likely it is to outcompete the others and survive (including the strategy to use fermentation even in the presence of oxygen in order to produce ethanol that inhibits growth of many other organisms). It might seem more economical to stop rRNA synthesis completely by repression of RNA pol I activity. However, complete repression of rDNA transcription is likely disadvantageous. Inhibition of RNA pol I transcription, e.g., by actinomycin D, leads to disruption of nucleolar morphology and diffusion of ribosome biogenesis factors [57,58]. In contrast, nutrients starvation or rapamycin treatment leaves nucleoli unchanged [57]. The maintenance of the nucleolus and its structure is intrinsically dependent on the ongoing ribosome biogenesis. We suggest that continued low transcription of rDNA followed by the A3 cleavage of 35S pre-rRNA, both of which occur in the nucleolus, allow cells to maintain nucleolar integrity and to keep high-effective, local concentration of ribosome biogenesis factors. Cells therefore remain poised to switch rapidly to exponential growth should the conditions improve. The capability to rapidly resume growth and compete for resources against other organisms provides an evolutionary advantage that greatly outweighs the energy loss required for sustained pre-rRNA synthesis.

In summary, we demonstrate that TORC1 and the CK2 kinase regulate ribosome biogenesis at the posttranscriptional level, independently of the Sch9p kinase branch. We identified Mak11p and Rrp5p as potential targets of CK2 or TORC1 in preribosomes. These findings provide a deep insight into the mechanisms by which cells regulate ribosome biogenesis in response to changing environmental conditions and contribute to our understanding of regulation of cellular processes in preparation for survival, such as entry to quiescence.

## Materials and methods

### Cell culture

The *S*. *cerevisiae* strains were grown in YPD, SDC-ura, SDC-his, or YPG (all from Formedium, United Kingdom) and harvested as described in the figures. Standard yeast techniques were used as in [59]. The tetracycline depletion strains were constructed and depleted as described in [60,65]. Strains used in this study are listed in S2 Table. For treatment with inhibitors, rapamycin was added from a stock in DMSO to final concentration 200 ng/ml. To inhibit CK2, 4,5,6,7-Tetrabromobenzotriazole (TBB) (Santa Cruz Biotech or Merck) was added from 10 mM stock in DMSO to final concentration 100 μM. In both cases, the control cultures were treated with DMSO only in the equivalent final concentration of DMSO.

### RNA extraction and northern blotting

Total RNA was extracted with guanidine, phenol-chloroform extractions, and precipitation as described [61]. Northern blotting and hybridization were performed as described [62], using the total RNA isolated from equivalent number of cells per lane. RNA was denatured with glyoxal and run overnight on 1% agarose-BTPE gel. The RNA was then electro-transferred on positively charged membrane and hybridized in Church buffer with end-labeled oligonucleotide probes (S3 Table). After, washing blots were exposed to phosphor imaging plates (Fuji), scanned on the Fuji FLA7000 imager (Fuji), and quantified with AIDA Image Analyzer software (Raytest, Germany). All experiments were done in two or more biological replicas.

### Pulse-chase

Pulse-chase experiments and quantification were performed as previously described [26]. Briefly, to exponential or postdiauxic shift cultures growing at 30°C in SD-Ura media, [5,6-^3^H]-Uracil was added for the time indicated. The chase was initiated by the addition of 100 mM Uracil. Samples of 1 ml volume were collected at the different time points, centrifuged, media removed, and pellets frozen. Total RNA was extracted, separated in 1.2% agarose-glyoxal gels, and transferred to nylon membrane. The membranes were exposed to Fuji BAS imaging plates and scanned on the FLA-7000 imager (Fuji). All experiments were done in two or more biological replicas.

### TAP purification and SILAC mass spectrometry

Cells were grown exponentially in SDC (light media) or after diauxic shift/rapamycin treatment in SDC containing ^13^C, ^15^N-L-Arginine and ^13^C, ^15^N-L-Lysine (heavy media). For diauxic shift, the cells were harvested at OD_600_ 2 and 4.5. For rapamycin or TBB treatment, the OD_600_ was 2.0. An equal number (ODs) of cells were harvested separately (to avoid exposure of the control cells to rapamycin/TBB, or vice versa of depleted cells to glucose), pellets were mixed, lysed, and 90S preribosomes were affinity purified through both affinity steps using Pwp2-FLAG-TEV-ProteinA as bait. For detection of phosphorylation sites, phosphopeptides were enriched on TiMAC. The proteins present in the purified Pwp2-associated preribosomes and their phosphorylation were determined by mass spectrometry at the FingerPrints Proteomics, Dundee), and raw data was analyzed by MaxQuant software [63,64] with default settings. The H/L ratios of individual proteins were normalized to the H/L ratio of the bait (which was thus set to 1). For phosphosites, the H/L ratios were normalized to H/L ratio of the protein abundance (all peptides quantified). All experiments were done in two or more biological replicas.

## Supporting information

S1 Fig Pre-rRNA processing switches from A2 to A3 pathway in 20 minutes during diauxic shift. (A) YMK118 was grown in SDC and (B) YMK118 was grown in YPD. Total RNA was isolated at the indicated OD_600_, and analyzed by Northern blotting. (C) Pre-rRNA processing in W303A and BY4741 strains. (D) Pre-rRNA processing in YMK118 exposed to oxidative stress by 0.2 mM Diamide at OD_600_ = 2. Northern blotting probes are as described in Fig 1A and 1B.
(TIF)

S2 Fig RNA composition of Pwp2-pre-ribosomes before and after diauxic shift. YMK118 cells expressing Pwp2-FLAG-TEV-ProteinA were grown to OD_600_ = 4 or OD_600_ = 10 respectively, lysed and pre-ribosomes purified on IgG sepharose beads. The bound RNA was analyzed by Northern blotting. Tot = total RNA, inp = input, elu = eluate.
(TIF)

S3 Fig Involvement of diverse factors in the A2 to A3 switch. Strains deleted for different factors (as indicated in the figure) were grown in YPD and harvested over time, total RNA was extracted and analyzed by Northern blotting using the A2-A3 probe.
(TIF)

S4 Fig The activity of PKA is not required for the switch. PKA analogue sensitive strain YAH475 was treated or not with 200nM 1NM-PP1 at 2.3 OD_600_. The parental strain YAH473 was used as a control. Yeast was then harvested at the indicated times, total RNA extracted and Northern blot performed.
(TIF)

S5 Fig Ribosomal protein mRNAs are co-downregulated with pre-rRNAs during the switch from A2 to A3 processing. RNA from various experiments was reloaded on denaturing polyacrylamide gels and analyzed by Northern blotting. It was then probed with 6 different mRNA probes (S9 Table). The RNA samples are from the following experiments: “YPD” from S1B Fig, “SDC” from S1A Fig, “Glucose” from Fig 1C, “Rapamycin” from Fig 3A, “TBB” from Fig 6A. “-”represents DMSO.
(TIF)

S6 Fig Depletion of Sch9 does not affect the A2 to A3 switch. (A) P_TetO7_::Sch9 strain expressing either wild type Sch9 or Sch9-2D3E or empty plasmid was depleted for Sch9 by addition of doxycycline. After the yeast went into diauxic shift, were diluted in fresh media and harvested after 10, 20, 30 and 60 minutes. Total RNA was extracted and Northern blot performed with A2-A3 probe. (B) At the first time point a sample from each strain was taken and subjected to Western Blot with an anti-HA antibody.
(TIF)

S7 Fig High resolution version of Fig 2D. (TIF)

S8 Fig H/L enrichment ratios of the small subunit ribosomal proteins (RPSs) in Pwp2-pre-ribosomes. (TIF)

S1 Table List of ribosome biogenesis factors with a change in phosphorylation identified in previous studies. (XLSX)

S2 Table Strains used in this study. (DOCX)

S3 Table Oligonucleotides used as hybridization probes. (DOCX)

S1 Data Numerical results of the proteomic analyses. (XLSX)

## Abbreviations

CARA: Constitutive Association of Rrn3 and A43
CURI: CK2, Utp22p, Rrp7p, and Ifh1p
H/L: Heavy/Light
FRB: FKBP−rapamycin-binding domain
iBAQ: intensity based absolute quantification
PHO85: phosphate metabolism 85
PKA: protein kinase A
rDNA: ribosomal DNA
RP: ribosomal protein
RPL: ribosomal protein of the large subunit
RPS: ribosomal protein of the small subunit
RNA pol I: RNA polymerase I
RNA pol II: RNA polymerase II
RNA pol III: RNA polymerase III
rRNA: ribosomal RNA
SNF1: sucrose non-fermenting 1
SDC: synthetic dextrose complete
TBB: Tetrabromobenzotriazole
Tor1: target of rapamycin 1
TORC1: target of rapamycin complex 1
YPD: yeast extract-peptone-dextrose
