# Supplementary figures and images for: Tor1 and CK2 kinases control a switch between alternative ribosome biogenesis pathways in a growth-dependent manner
Source: PLoS Biol. 2017 Mar 10;15(3):e2000245. doi: 10.1371/journal.pbio.2000245 (PMC5345768; doi:10.1371/journal.pbio.2000245)

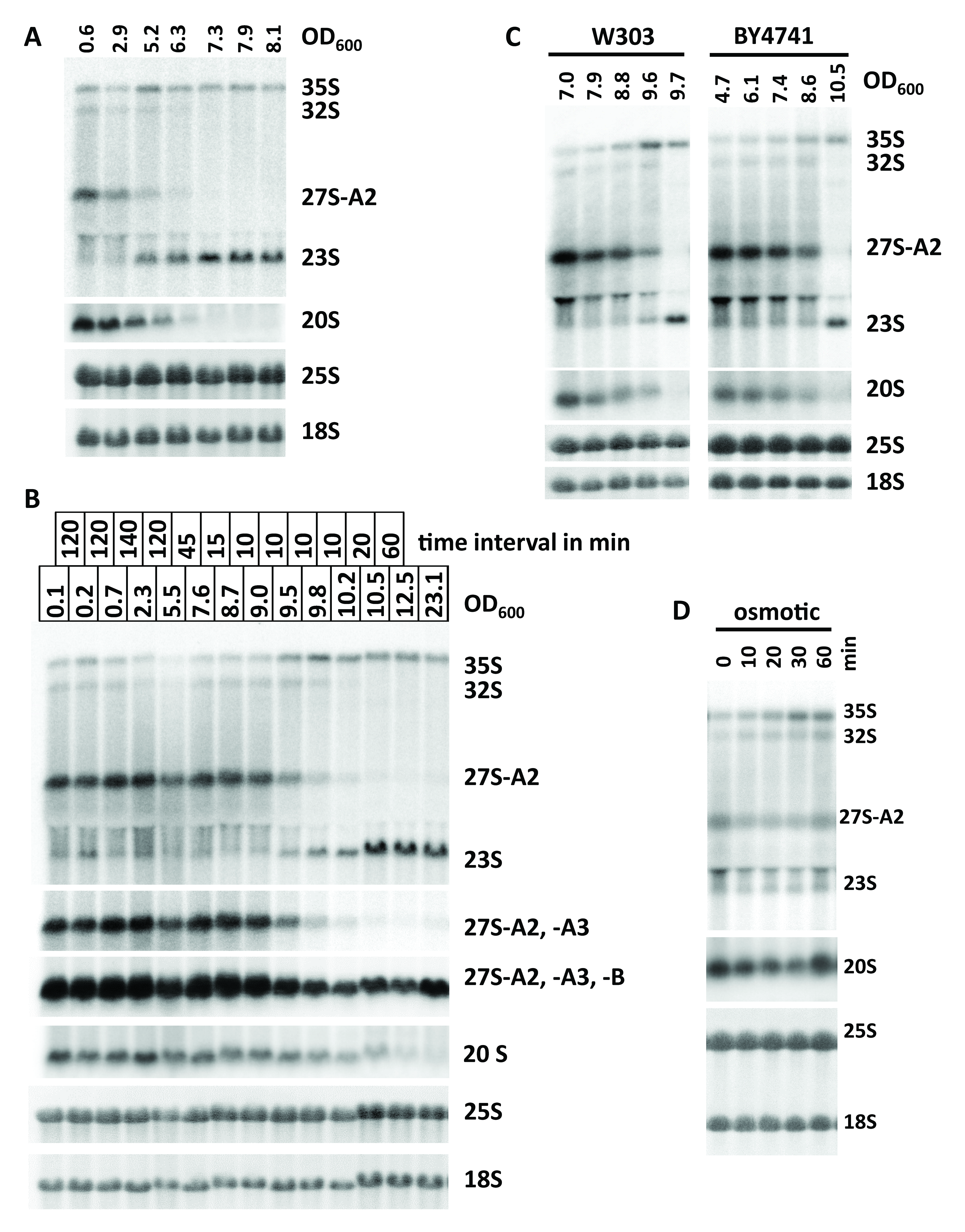

Supplement: S1 Fig — (A) YMK118 was grown in SDC and (B) YMK118 was grown in YPD. Total RNA was isolated at the indicated OD600, and analyzed by Northern blotting. (C) Pre-rRNA processing in W303A and BY4741 strains. (D) Pre-rRNA processing in YMK118 exposed to oxidative stress by 0.2 mM Diamide at OD600 = 2. Northern blotting probes are as described in Fig 1A and 1B. (TIF) [file pbio.2000245.s001.tif]

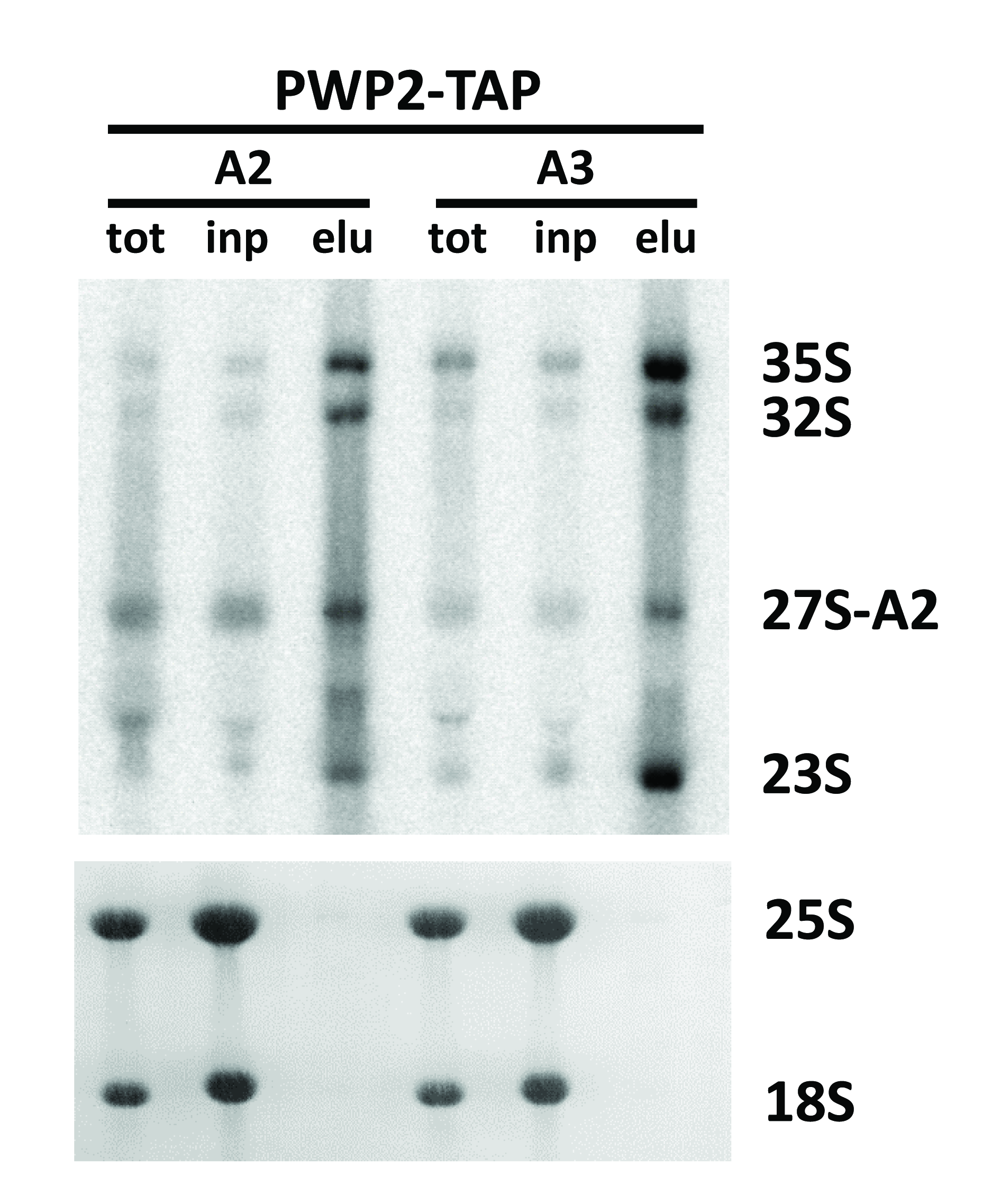

Supplement: S2 Fig — YMK118 cells expressing Pwp2-FLAG-TEV-ProteinA were grown to OD600 = 4 or OD600 = 10 respectively, lysed and pre-ribosomes purified on IgG sepharose beads. The bound RNA was analyzed by Northern blotting. Tot = total RNA, inp = input, elu = eluate. (TIF) [file pbio.2000245.s002.tif]

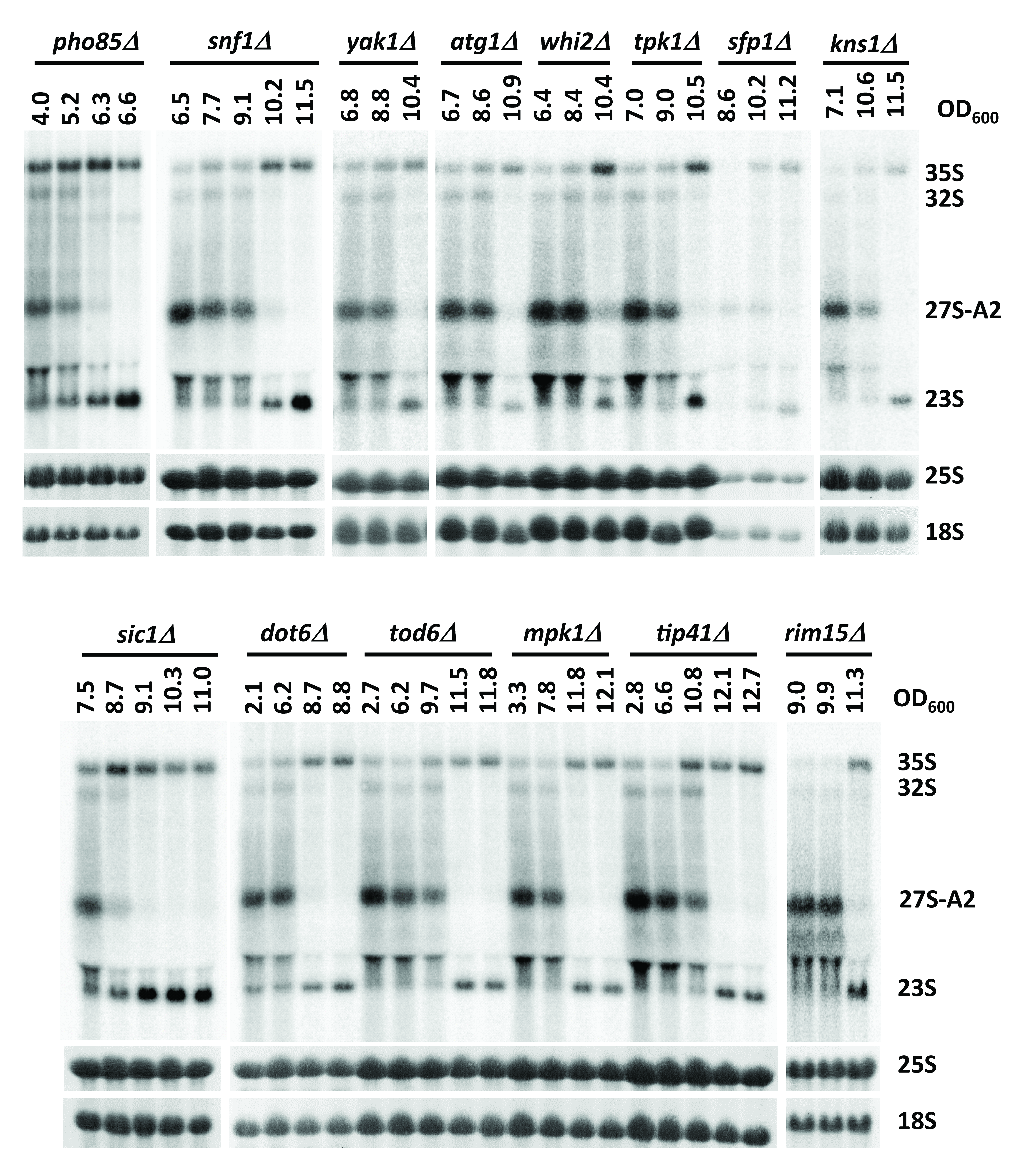

Supplement: S3 Fig — Strains deleted for different factors (as indicated in the figure) were grown in YPD and harvested over time, total RNA was extracted and analyzed by Northern blotting using the A2-A3 probe. (TIF) [file pbio.2000245.s003.tif]

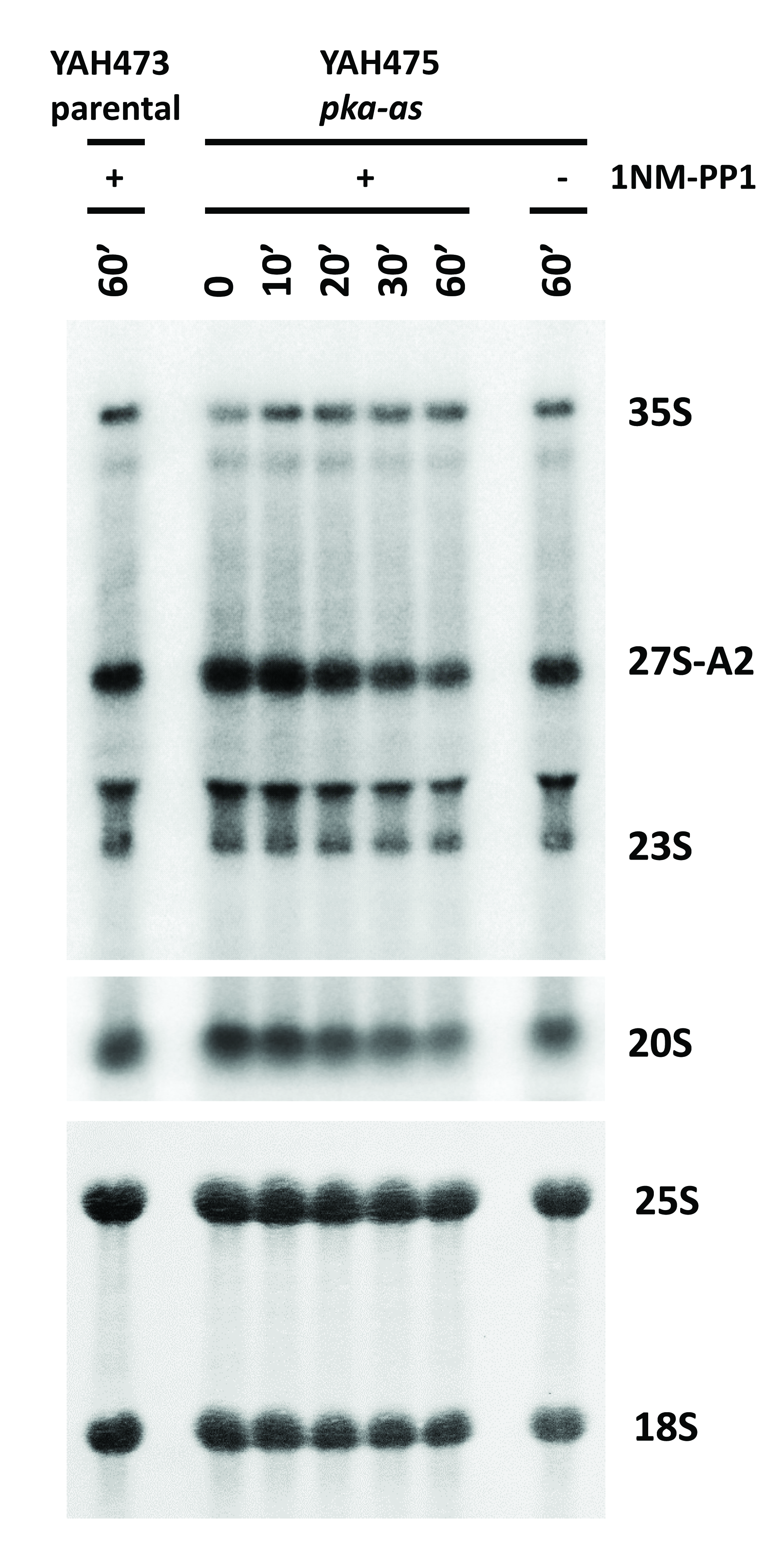

Supplement: S4 Fig — PKA analogue sensitive strain YAH475 was treated or not with 200nM 1NM-PP1 at 2.3 OD600. The parental strain YAH473 was used as a control. Yeast was then harvested at the indicated times, total RNA extracted and Northern blot performed. (TIF) [file pbio.2000245.s004.tif]

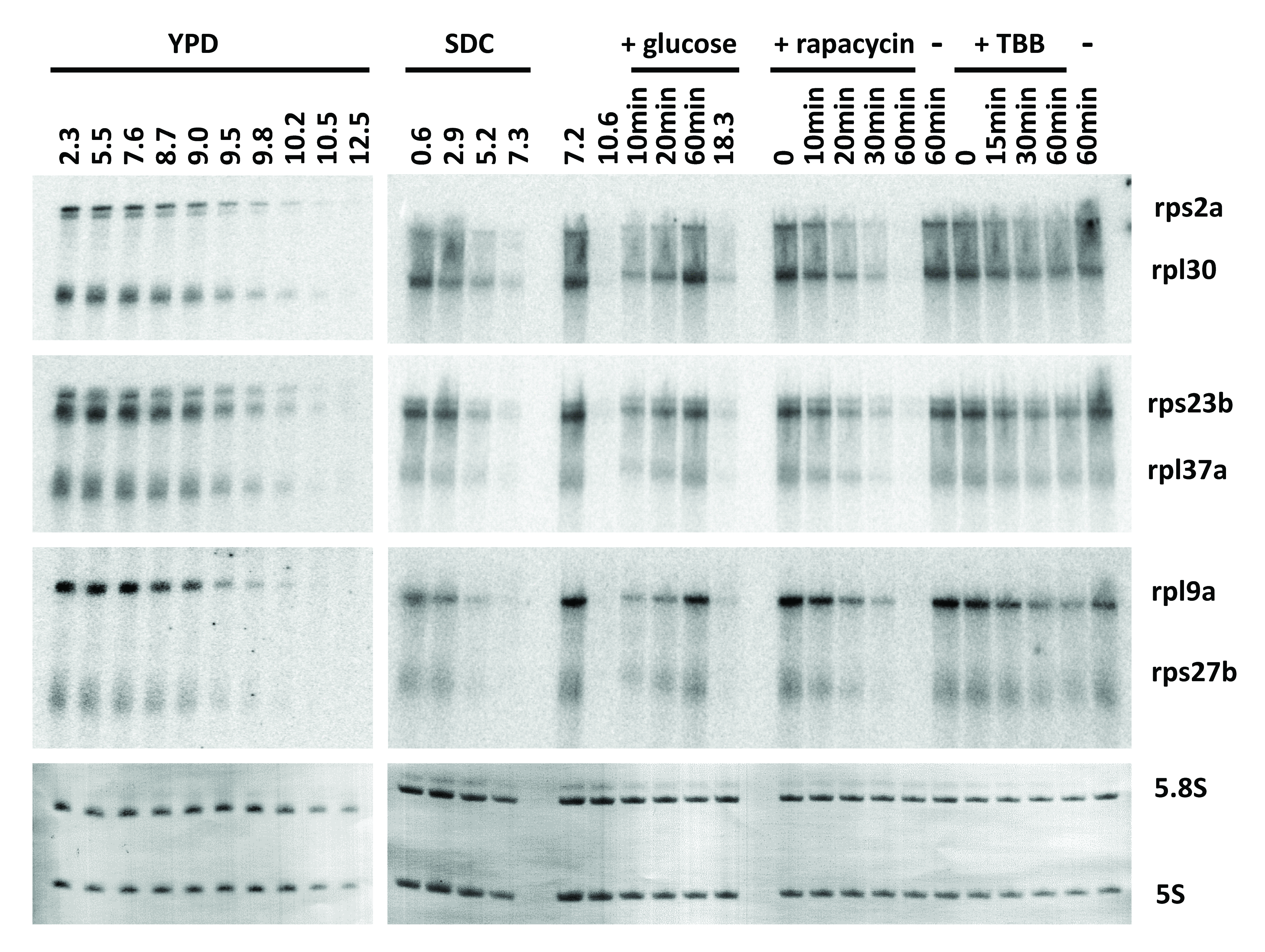

Supplement: S5 Fig — RNA from various experiments was reloaded on denaturing polyacrylamide gels and analyzed by Northern blotting. It was then probed with 6 different mRNA probes (S9 Table). The RNA samples are from the following experiments: “YPD” from S1B Fig, “SDC” from S1A Fig, “Glucose” from Fig 1C, “Rapamycin” from Fig 3A, “TBB” from Fig 6A. “-”represents DMSO. (TIF) [file pbio.2000245.s005.tif]

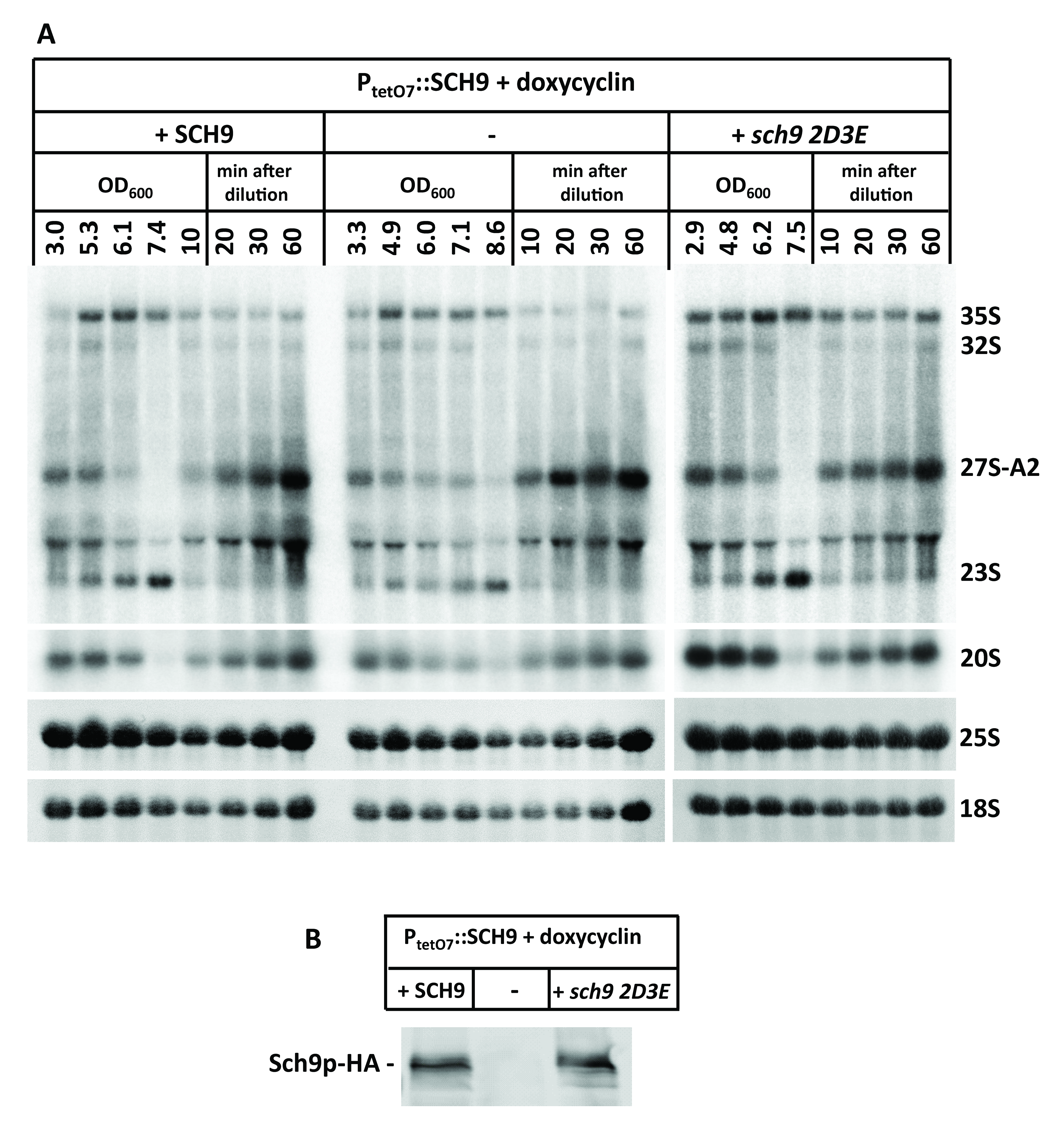

Supplement: S6 Fig — (A) PTetO7::Sch9 strain expressing either wild type Sch9 or Sch9-2D3E or empty plasmid was depleted for Sch9 by addition of doxycycline. After the yeast went into diauxic shift, were diluted in fresh media and harvested after 10, 20, 30 and 60 minutes. Total RNA was extracted and Northern blot performed with A2-A3 probe. (B) At the first time point a sample from each strain was taken and subjected to Western Blot with an anti-HA antibody. (TIF) [file pbio.2000245.s006.tif]

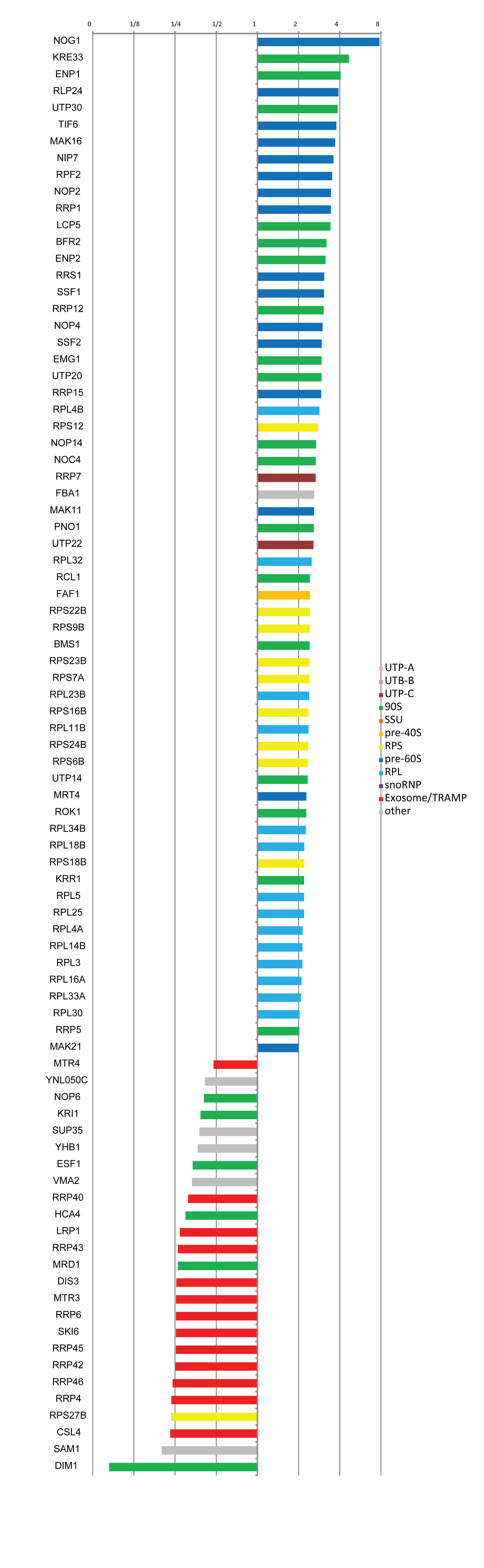

Supplement: S7 Fig — (TIF) [file pbio.2000245.s007.tif]

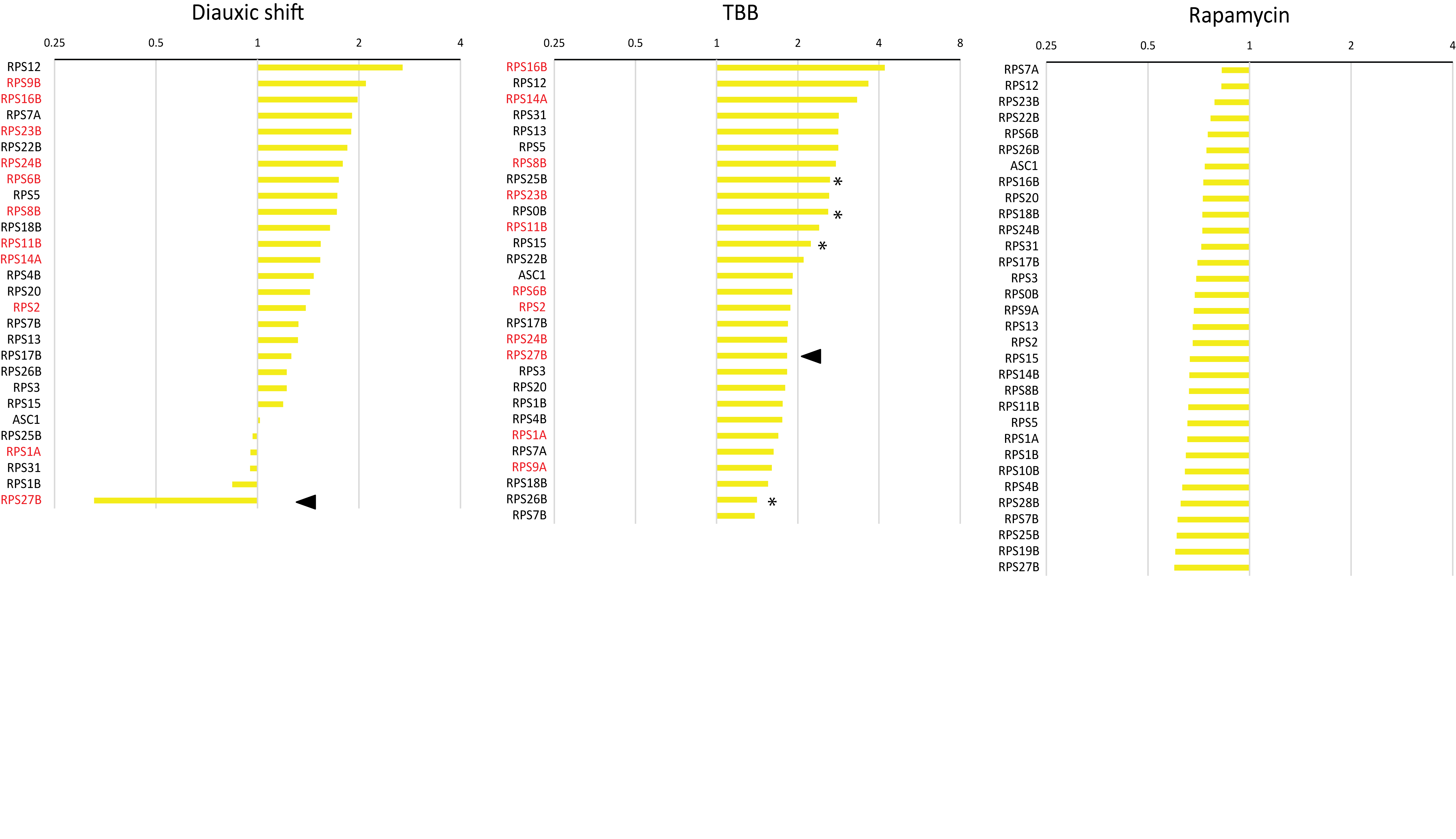

Supplement: S8 Fig — (TIF) [file pbio.2000245.s008.tif]
