# Supplementary material for: Tor1 and CK2 kinases control a switch between alternative ribosome biogenesis pathways in a growth-dependent manner
Source: PLoS Biol. 2017 Mar 10;15(3):e2000245. doi: 10.1371/journal.pbio.2000245 (PMC5345768; doi:10.1371/journal.pbio.2000245)
Supplement: S2 Table — (DOCX) [file pbio.2000245.s010.docx]

| **CEN PK2 1C** | MATa, his3∆1, leu2-3,112, trp1-289, ura3-52, MAL2-8C, SUC2 |  |
| --- | --- | --- |
| **YMK118** | CEN PK2 1C, lys2:: tTA, ura3::PCMVtetR'-SSN6 URA3-K.I. | [65] |
| **W303** | MATa, ade2-1, his3-11,15, leu2-3,112, trp1-1, ura3-1, can1-100 |  |
| **BY4741** | MATa; his3Δ1; leu2Δ0; met15Δ0; ura3Δ0 |  |
| **pwp2-TAP** | YMK118 Arg4::Kan, pwp2-flag-tev-proteinA | this study |
| **NOY892** | MATa; ade2-1; ura3-1; trp1-1; leu2-3,112; his3-11; can1-100; rdnDD::HIS3 + plasmid pNOY130 (GAL7-35S+5S, URA3) | [36] |
| **HHY168** | alpha; W303 tor1-1 fpr1::NAT RPL13A-2XFKB12::TRP1 | [37] |
| **BEN135** | HHY168 Rpa135-frb::KanMX6 | [38] |
| **YDS2** | A; leu2-3,112 his3-11,15 ura3-1 ade2-1 trp1-1 can1-100 (Rothstein, 1989) |  |
| **YVM47 (CARA)** | YDS2, rpa43::hyg rrn3::hyg +pGEN with Rpa43-rrn3 fusion | [39] |
| **YAH473** | alpha; W303 HHF2-mCherry::HphMX4 (gift from D. Shore/R. Loewith) |  |
| **YAH475 pka-as** | alpha; W303 tpk1-as tpk2-as tpk3-as HHF2-mCherry::HphMX4 (gift from D. Shore/R. Loewith) |  |
| ***Δsch9*** | BY4741; MATa; ura3Δ0; leu2Δ0; his3Δ1; met15Δ0; YHR205w::kanMX4 | Euroscarf Y07797 |
| **Sch9 2D3E** | YMK118 + plasmid pRS413-SCH9-2D3E (Claudio De Virgilio) | this study |
| **tet-Sch9 + WT** | YMK118, PtetO7-Ubi-Leu::Sch9 + pRS413-SCH9 | this study |
| **tet-Sch9** | YMK118, PtetO7-Ubi-Leu::Sch9 + pRS413 | this study |
| **tet-Sch9 + 2D3E** | YMK118, PtetO7-Ubi-Leu::Sch9 + pRS413-SCH9 2D3E | this study |
| **tap42-11** | BY4741; MATa; ura3Δ0; leu2Δ0; his3Δ1; met15Δ0; tap42-11:kanMX | Euroscarf Y41118 |
| **Ras2 V19** | YMK118 + plasmid p751 YEp213-RAS2-Val19 (gift from Claudio De Virgilio) |  |
| ***Δrrp6*** | BY4741 (YMK5); deletion Rrp6::kanMX4 | Euroscarf Y01777 |
| ***Δrrp47*** | W303 alpha, deletion rrp47::His3 Mx6 (gift from Mathias Toms/Ed hurt) |  |
| ***Δtrf4*** | trf4Δ;BY4741; MATa; ura3Δ0; leu2Δ0; his3Δ1; met15Δ0; YOL115w::kanMX4 | Euroscarf Y06265 |
| ***Δxrn1*** | xrn1Δ; BY4741; MATa; ura3Δ0; leu2Δ0; his3Δ1; met15Δ0; YGL173c::kanMX4 | Euroscarf Y04540 |
| ***Δpho85*** | BY4741, MATa; ura3Δ0; leu2Δ0; his3Δ1; met15Δ0; YPL031c(Pho85)::kanMX4 | Euroscarf Y02797 |
| ***Δsnf1*** | YMK1031: YMK118; Snf1::KanMX4 | this study |
| ***Δyak1*** | BY4741; MATa; ura3Δ0; leu2Δ0; his3Δ1; met15Δ0; YJL141c::kanMX4 | Euroscarf Y07006 |
| ***Δatg1*** | BY4741; MATa; ura3Δ0; leu2Δ0; his3Δ1; met15Δ0; YGL180w::kanMX4 | Euroscarf Y04547 |
| ***Δwhi2*** | BY4741; MATa; ura3Δ0; leu2Δ0; his3Δ1; met15Δ0; YOR043w::kanMX4 | Euroscarf Y01819 |
| ***Δtpk1*** | BY4741; MATa; ura3Δ0; leu2Δ0; his3Δ1; met15Δ0; YJL164c::kanMX4 | Euroscarf Y01261 |
| ***Δsfp1*** | BY4741; MATa; ura3Δ0; leu2Δ0; his3Δ1; met15Δ0; YLR403w::kanMX4 | Euroscarf Y05312 |
| ***Δkns1*** | BY4741; MATa; ura3Δ0; leu2Δ0; his3Δ1; met15Δ0; YGL173c::kanMX4 | Euroscarf Y04540 |
| ***Δsic1*** | BY4741; MATa; ura3Δ0; leu2Δ0; his3Δ1; met15Δ0; YLR079w::kanMX4 | Euroscarf Y02690 |
| ***Δdot6*** | BY4741; MATa; ura3Δ0; leu2Δ0; his3Δ1; met15Δ0; YER088c::kanMX4 | Euroscarf Y06393 |
| ***Δtod6*** | BY4741; MATa; ura3Δ0; leu2Δ0; his3Δ1; met15Δ0; YBL054w::kanMX4 | Euroscarf Y03080 |
| ***Δmpk1*** | BY4741; MATa; ura3Δ0; leu2Δ0; his3Δ1; met15Δ0; YHR030c::kanMX4 | Euroscarf Y00993 |
| ***Δtip41*** | BY4741; MATa; ura3Δ0; leu2Δ0; his3Δ1; met15Δ0; YPR040w::kanMX4 | Euroscarf Y05459 |
| ***Δrim15*** | YMK1015: YMK118; Rim15::KanMX4 | this study |
