# Supplementary material for: Tor1 and CK2 kinases control a switch between alternative ribosome biogenesis pathways in a growth-dependent manner
Source: PLoS Biol. 2017 Mar 10;15(3):e2000245. doi: 10.1371/journal.pbio.2000245 (PMC5345768; doi:10.1371/journal.pbio.2000245)
Supplement: S3 Table — (DOCX) [file pbio.2000245.s011.docx]

| **20S** | **002** | CGGTTTTAATTGTCCTA |
| --- | --- | --- |
| **A2-A3** | **800** | GCAAAGATATGAAAACTCCAC |
| **A3-B1** | **863** | GTTCCAGTTACGAAAATTCTTGT |
| **7S** | **006** | GGCCAGCAATTTCAAGTTA |
| **RPS2** |  | CACCAGCAACTTCCTTG |
| **RPS23B** |  | GCAGAGTTAGGTTGCTTG |
| **RPS27B** |  | CAGTGGTGATGTTCAAAC |
| **RPL9A** |  | TGCTTCAAGTTCTTGGTC |
| **RPL30** |  | CAGAGTCACCAGCTTCC |
| **RPL37A** |  | CTTGAATCTTCTTGAAACG |
